# Investigation for the easy and efficient synthesis of 1*H*-benzo[*d*][1,3]oxazine-2,4-diones†

**DOI:** 10.1039/d5ra04014k

**Published:** 2025-08-04

**Authors:** Nikolaos Mitsostergios, Vasileios Athanasopoulos, Spyridon Mourtas

**Affiliations:** a Department of Chemistry, University of Patras 26510 Rio Patras Greece s.mourtas@upatras.gr

## Abstract

We investigated a two-step approach for the easy and efficient synthesis of 1*H*-benzo[*d*][1,3]oxazine-2,4-diones starting from 2-aminobenzoic acids, using the urethane type fluorenylmethyloxycarbonyl (Fmoc), benzyloxycarbonyl (Cbz) and ethyloxycarbonyl (EtOCO) groups as the source of carbonyloxy group, and thionyl chloride to promote activation, cyclization and 1*H*-benzo[*d*][1,3]oxazine-2,4-diones formation.

## Abstract 2

We report the synthesis of 1*H*-benzo[*d*][1,3]oxazine-2,4-diones from 2-aminobenzoic acids, using fluorenylmethyloxycarbonyl (Fmoc), benzyloxycarbonyl (Cbz) and ethyloxycarbonyl (EtOCO) groups as the source of carbonyloxy group, and thionyl chloride.

## Graphical abstract

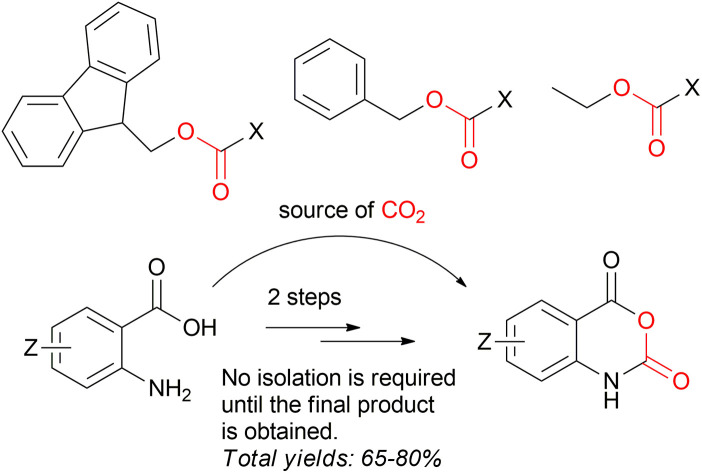

## Introduction

**Fig. 1:**
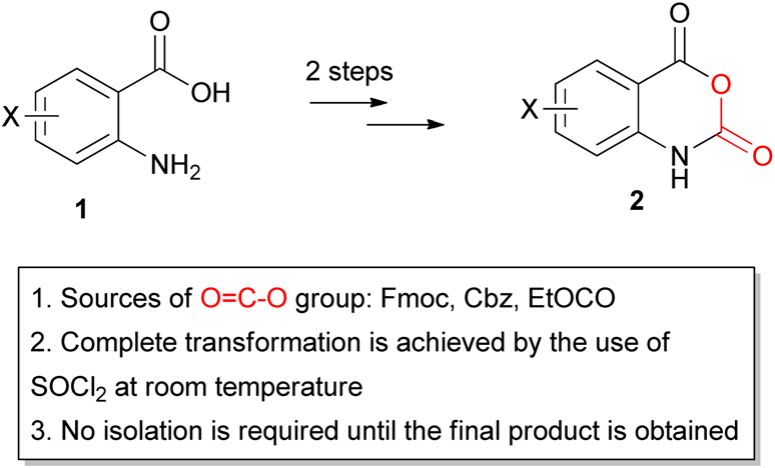
General structure of the targeted 1*H*-benzo[*d*][1,3]oxazine-2,4-diones 2 from 2-aminobenzoic acids 1, and main aspects of this investigation.

1*H*-Benzo[*d*][1,3]oxazine-2,4-diones represent a class of heterocyclic compounds characterized by their unique structural framework (Fig. 1), allowing them to interact with various biological targets. For example, they have been shown to be potent hepatitis C virus (HCV) inhibitors,^1^ butyrylcholinesterase (BChE) inhibitors,^2^ and histone acetyltransferases (HATs) inhibitors.^3^ In addition, they have been reported as antiallergic,^4^ antitumor,^5^ antipsychotic,^6^ and antimycobacterial agents.^7^ The ability of these compounds to release carbon dioxide also allows their use in the synthesis of a wide variety of heterocyclic molecules,^8^ while they have been used as precursors in other bioactive molecules. As an example, isatoic anhydride, the most simple 1*H*-benzo[*d*][1,3]oxazine-2,4-dione, has been used as a precursor for the synthesis of methaqualone and related 4-quinazolinone-based pharmaceutical drugs.^9^ Other 1,3-benzooxazine-2,4-diones have been used in the synthesis of several potent bronchodilators, antipsychotic, anti-oxidant, anti-inflammatory, and antimicrobial agents.^10–15^

Regarding their synthesis, 1*H*-benzo[*d*][1,3]oxazine-2,4-diones have been synthesized from several starting materials, including 2-aminobenzoic acid (anthranilic acid), 2-(azidocarbonyl)benzoic acid, phthalamic acid, indoline-2,3-dione (isatin), benzo[*c*]isoxazole, phthalic anhydride, phtalimide. In all these cases, the applied methodologies require oxidizing agents such as peroxyacids and peroxides, highly toxic and poisonous solvents (*e.g.* phosgene), catalysts (such as diselenides), toxic metal catalysts (Pd-catalyst), explosive reagents (such as trimethylsylilazide), or microwave-assisted methodologies and carbon dioxide. In addition, long reaction times and high temperatures have been reported, while in most cases the reaction yields are low to moderate.^10,11,16–20^ In other cases, 2-chloromethylpyridinium iodide was reacted with *tert*-butyloxycarbonyl substituted 2-aminobenzoic acids to afford 1*H*-benzo[*d*][1,3]oxazine-2,4-diones after acidic treatment,^21^ while the use of oxalyl chloride with ethoxycarbonyl substituted 2-aminobenzoic acids under reflux conditions,^22,23^ and the use of thionyl chloride with methoxy/ethoxycarbonyl substituted 2-aminobenzoic acids have also been applied^24,25^, but the use of moderate to high temperatures were reported and the studies were not systematic. On large scale, isatoic anhydride is considered to be synthesized by the reaction of phthalimide with sodium hypochlorite in the presence of sodium hydroxide.^26^ However, if the reaction temperature is not controlled the yield is significantly reduced, while large effluents of wastewater are produced.^11^

The great potential of 1*H*-benzo[*d*][1,3]oxazine-2,4-diones to be used in organic synthesis and their interesting biological activities led us to explore their synthesis *via* easy methods that ensure high yields and purity, avoiding long reaction times, high temperatures or reflux conditions, non-trivial, toxic or explosive reagents/solvents, *etc.* To this end, we also considered that industrial methods often wish to reduce the required energy and minimize the purification steps, thus improving the yield of reactions and achieving efficient production. By considering these needs, we investigated a two-step approach for the synthesis of compounds 2 starting from 2-aminobenzoic acids 1 (Fig. 1) by using the urethane type fluorenylmethyloxycarbonyl (Fmoc), benzyloxycarbonyl (Cbz) and ethyloxycarbonyl (EtOCO) groups as the source of carbonyloxy group and thionyl chloride (a solvent widely used – usually preferred in pharmaceutical industry) as an activating agent to enable cyclization.

To our knowledge, there is no such comparative study between Fmoc/Cbz/EtOCO groups as the source of carbonyloxy group and thionyl chloride, while our experimental findings allowed us to propose an easy two-step procedure for the introduction of the urethane groups and subsequent activation/cyclization and final transformation by thionyl chloride at room temperature. In addition, our experimental strategy allowed us to provide insights into the mechanism involved in this transformation. As a result, a general method for the easy and efficient synthesis of 1*H*-benzo[*d*][1,3]oxazine-2,4-diones is proposed.

## Results and discussion

### 1*H*-benzo[*d*][1,3]oxazine-2,4-diones from Fmoc-2-aminobenzoic acids

In our efforts to synthesize the *o*, *m*, and Fmoc-*p*-aminobenzoyl chlorides through the reaction of the corresponding carboxylic acids with thionyl chloride (SOCl_2_),^27^ we realized that the reaction of 3a and 3b with SOCl_2_ resulted in chemical instability. This finding forced us to further investigate this observation.

**Scheme 1:**
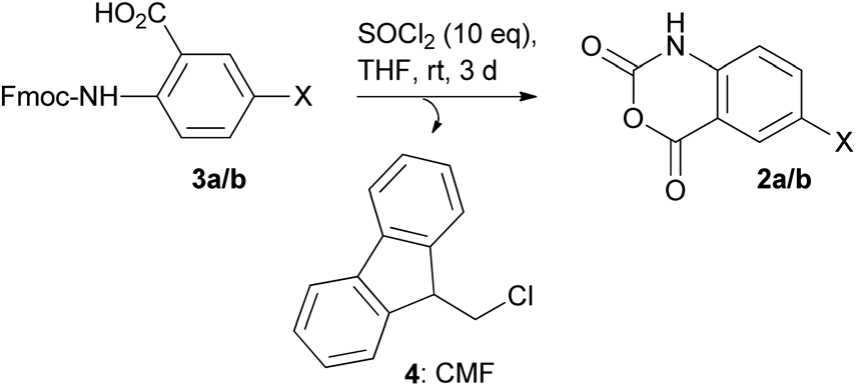
Synthesis of 2a/b from 3a/b; X = H (3a), CH_3_ (3b).

For this, we initially used HPLC analysis (and ESI-MS) to monitor the reaction progress, where we identified that the reaction of Fmoc-2-aminobenzoic acids 3a/b with SOCl_2_ finally afforded 2a/b and another product which was identified-in both cases- as 9-(chloromethyl)-9*H*-fluorene (CMF) 4 (Scheme 1).

In Fig. S1A1–A6† we present a representative HPLC chromatogram during the reaction of 3a with 10-fold molar excess of SOCl_2_ in THF at room temperature (22–24 °C), and in Fig. S2† we present the HPLC analysis during the reaction of 3b with SOCl_2_ (under the same conditions). As can be seen, besides the peak that corresponds to the starting compound 3a (or 3b), a new peak was identified (marked with an asterisk), which gradually decreased as the reaction progressed, to finally afford 2a/b and 4 as the only products. Chemical characterization/identification of 4 was achieved by performing semi-preparative HPLC for both transformations (3a to 2a, and 3b to 2b), and the collected fragments (corresponding to 4) were free-dried and subjected to ^1^H and ^13^C-NMR analysis (Fig. S3†) by which the formation of 4 was proved. Both products 2a and 2b were easily isolated in high yields (>97% confirmed by NMR and HPLC analysis). The replacement of THF with DCM gave the same results, while the reaction of 3a with a 5-fold molar excess of SOCl_2_ in THF at room temperature (22–24 °C) was not sufficient to allow complete transformation (Fig. S1B1–B5†).

**Scheme 2:**
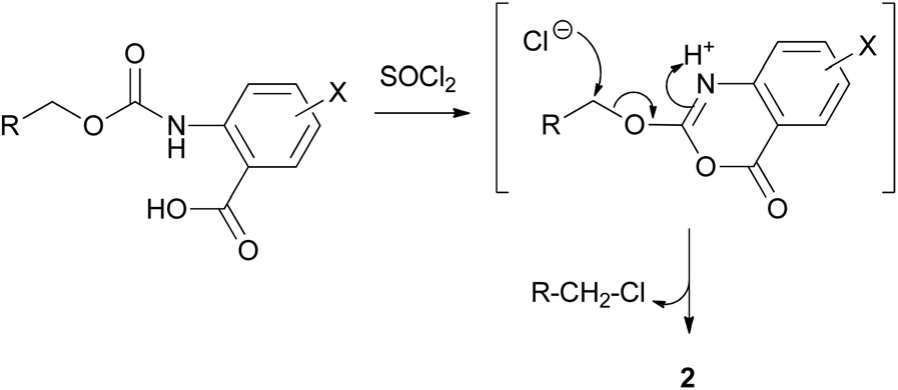
The use of SOCl_2_ enables ring closure to 2-alkyloxy-4*H*-3,1-benzo[*d*][1,3]oxazin-4-one and subsequent nucleophilic attack to finally afford RCH_2_Cl and 2.

Based on these observations, we rationalized that the use of SOCl_2_ enables activation of the carboxylic acid group, followed by nucleophilic attack of the neighboring carbamate oxygen to the activated carboxylic acid group, enabling ring closure to 2-alkyloxy-4*H*-3,1-benzo[*d*][1,3]oxazin-4-one to finally liberate 2 and 4 through the nucleophilic attack of chloride ions to the fluorenyl-methylene carbon atom (Scheme 2; R = fluorenyl; R-CH_2_–O–CO = Fmoc).

Similar base catalyzed mechanisms have been described in literature for similar substrates and activating agents.^28–30^ In our case, the activating agent responsible for the initiation of the reported transformation is SOCl_2_, allowing the formation of 2 and the release of 4, which was clearly identified as the only released product bearing the 9*H*-fluorenyl-methyl group.

To further prove our hypothesis for the active involvement of SOCl_2_ in this transformation, we replaced SOCl_2_ with TFA (1%, 50%, 90% v/v) in THF (and also in DCM), to test whether an acid-catalyzed transformation could be involved, driven by the nucleophilic attack of the carbamate oxygen to the neighboring activated carbonyl carbon of the protonated carboxylic acid group, where no subsequent cyclization or any other instabilities were noticed (regardless of the acid concentration that was used). This finding supported our hypothesis for the suggested reaction pathway, which is a combination of the initial activation of the carboxylic acid group by SOCl_2_ and the position (*ortho*) of the Fmoc-amino group, enabling the intramolecular cyclization and formation of a 6-membered ring, apparently thermodynamically favored, and a good leaving group, allowing the subsequent nucleophilic substitution by chloride anions.

In order to investigate the new formed peak that was seen in the HPLC chromatogram (marked with an asterisk) (Fig. S1 and S2†), we performed the following two efforts: (a) we performed semi-preparative HPLC, and the collected peak (marked with an asterisk in Fig. S1 and S2†), was lyophilized and subjected to NMR analysis. However, by this method, only 3a/b were identified (by ESI-MS and NMR), which was attributed (and later confirmed) to the hydrolysis of the proposed intermediate throughout the isolation/lyophilization process; (b) then, we attempted to investigate the reaction by real time NMR, following the reaction of 3a with SOCl_2_ (in CDCl_3_). However, the very low solubility of 3a (and 2a) in CDCl_3_ did not allow us to obtain the required data. Nevertheless, although we were not able to confirm the new peak that was evidenced by the HPLC analysis, nor were we able to directly link it with 2-alkyloxy-4*H*-3,1-benzo[*d*][1,3]oxazin-4-one, the proposed reaction pathway is rather rational due to the formation of 4 (and 2a/b), the only final products observed, and was further supported by replacing the fluorenylmethyloxycarbonyl (Fmoc) group with benzyloxycarbonyl (Cbz or Z) and ethoxycarbonyl (EtOCO) groups.

### 1*H*-Benzo[*d*][1,3]oxazine-2,4-diones from Cbz-2-aminobenzoic acids

**Scheme 3:**
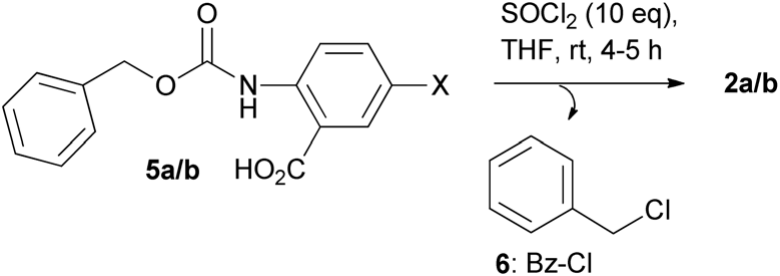
Synthesis of 2a/b from 5a/b; X = H (5a), CH_3_ (5b).

In the second set of experiments, we initially synthesized the corresponding 2-((benzyloxycarbonyl)amino)benzoic acids: Cbz-2-aminobenzoic acid (5a) and Cbz-2-amino-5-methylbenzoic acid (5b), by using methods that have been reported in literature, where a mixture of Na_2_CO_3_/NaHCO_3_ is used to maintain pH during the reaction of 2-aminobenzoic acids with benzyl chloroformate in water/acetone (1 : 4).^31^5a/b were further reacted with SOCl_2_ (10 molar excess) at room temperature (22–24 °C), and the reaction progress was followed by HPLC analysis (Fig. S4A1–A8 and S5†) (and ESI-MS), where the expected 2a/b and benzyl chloride 6 were identified (Scheme 3). In this case (compared with the Fmoc group), the formation of 2a/b was completed faster (within 5–7 h at room temperature) and both 2a/b were easily isolated in high yields (>97% as confirmed by NMR and HPLC analysis). Treatment of 5a with a 5-fold molar excess of SOCl_2_ resulted in a slower transformation (around 12 h for 2a) (Fig. S4B1–B6†), while in the case of a 2-fold molar excess of SOCl_2_ the transformation was not completed (Fig. S4C1–C5†).

In addition, the solubility of 5a in CDCl_3_ allowed us to follow its reaction progress with SOCl_2_ by NMR. By this experiment, we were able to clearly identify the formation of benzyl chloride 6 (Fig. S6A and B†) as the main product of this reaction, which is compatible and further supports the suggested reaction pathway. In addition, the replacement of the Fmoc group with the Cbz group seems to allow the faster formation of 2a/b and 6, since, under the same conditions tested (room temperature; 22–24 °C), the formation of 2a/b was almost completed within 4–5 h (Fig. S4–S5, S1 and S2†). The use of a 2-fold molar excess of SOCl_2_ confirmed the incomplete transformation monitored by hplc analysis (Fig. S6C and D†).

### 1*H*-Benzo[*d*][1,3]oxazine-2,4-diones from EtOCO-2-aminobenzoic acids

**Scheme 4:**
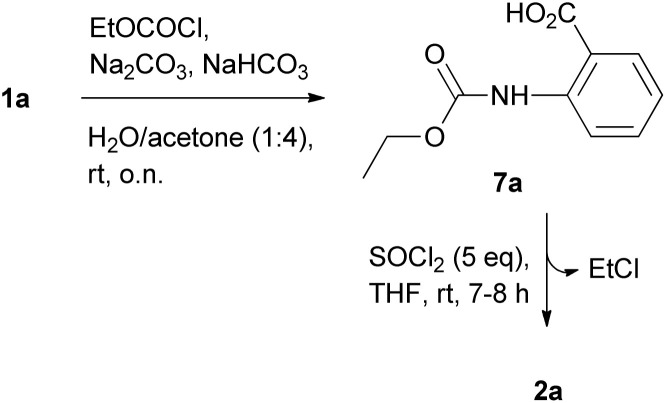
1a–1e: Synthesis of 2a from 1a.

The replacement of Cbz- with EtOCO-group as the source of carbonyloxy group in the desired 1*H*-benzo[*d*][1,3]oxazine-2,4-diones further supported the findings. Initially, we prepared the 2-((ethyloxycarbonyl)amino)benzoic acid (EtOCO-2-aminobenzoic acid) 7a by the reaction of ethoxycarbonyl chloride (EtOCOCl) with 2-aminobenzoic acid 1a using Na_2_CO_3_/NaHCO_3_ in water/acetone (1 : 4) (Scheme 4),^31^ and we followed the reaction of 7a with SOCl_2_ by HPLC analysis.

Four different reaction conditions (regarding SOCl_2_ molar excess and reaction temperature) were used: (a) 10-fold molar excess at 16–17 °C; (b) 10-fold molar excess at 22–24 °C; (c) a 5-fold molar excess at 22–24 °C and (d) a 2-fold molar excess at 22–24 °C. The HPLC chromatograms (during the reaction progress) are presented in Fig. S7–S10,† where, in all cases, besides the peak that corresponds to the starting compound 7a a new peak was formed (marked with an asterisk), which gradually decreased as the reaction progressed to the formation of 2a. In addition, it became evident that the cyclization process was almost completed within 4–5 h in the 10-fold molar excess of SOCl_2_ (Fig. S8†), and within 7–8 h in the 5-fold molar excess (Fig. S9†), while in case of the 2-fold molar excess, the cyclization reaction was implemented in around 50% (Fig. S10†).

In addition, although 7a was not completely dissolved in CDCl_3_, it was rapidly dissolved upon treatment with SOCl_2_, which allowed us to follow the reaction progress by NMR (Fig. S11A and B†), where the transformation of 7a to 2a through the release of ethyl chloride (EtCl) was clearly evidenced. The use of a 2-fold molar excess of SOCl_2_ confirmed the incomplete transformation monitored by hplc analysis also in this case (Fig. S11C and D†).

Comparing the reaction rate for the three different sources of carbonyloxy group (Fmoc, Cbz, EtOCO), it becomes evident that it follows the order EtOCO ≈ Cbz > Fmoc-group, possibly as a result of the lower steric hindrance in the case of EtOCO and Cbz groups compared to the bulky Fmoc group, allowing an easier nucleophilic attack on the methylene group of 2-alkyloxy-4*H*-benzo[*d*][1,3]oxazin-4-one core (to finally afford RCH_2_Cl and 1*H*-benzo[*d*][1,3]oxazine-2,4-dione, a good leaving group). Notably, no competing elimination reaction to the formation of 9-methylene-9*H*-fluorene (in the case of Fmoc-2-aminobenzoic acids) or ethylene (in the case of EtOCO-2-aminobenzoic acids) were observed.

**Scheme 5:**
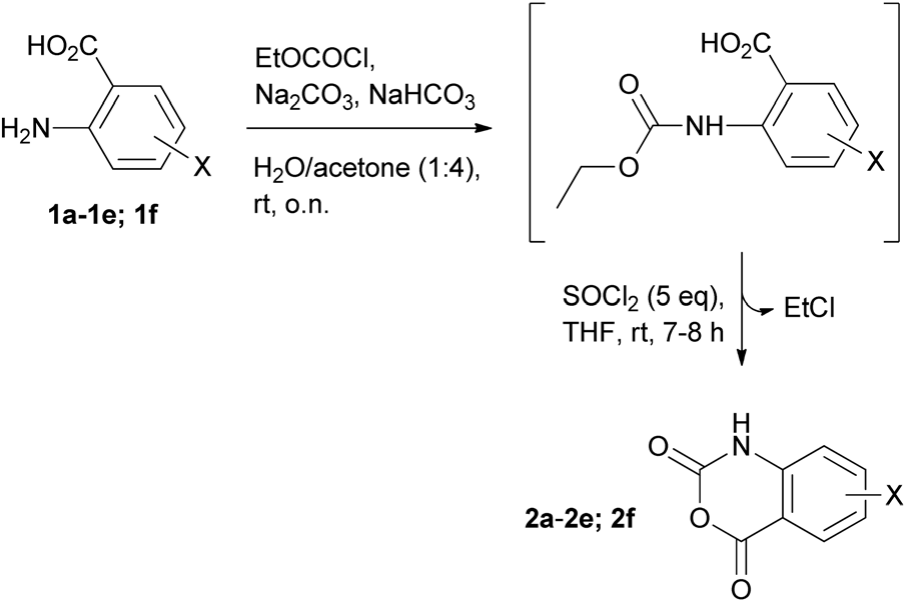
Proposed method for the synthesis of 2a–f from 1a–f; 1a–e: X = H (1a), 5-CH_3_ (1b), 5-Cl (1c), 4-NO_2_ (1d), 5-NO_2_ (1e); 1f: 3-amino-2-naphtoic acid.

**Fig. 2:**
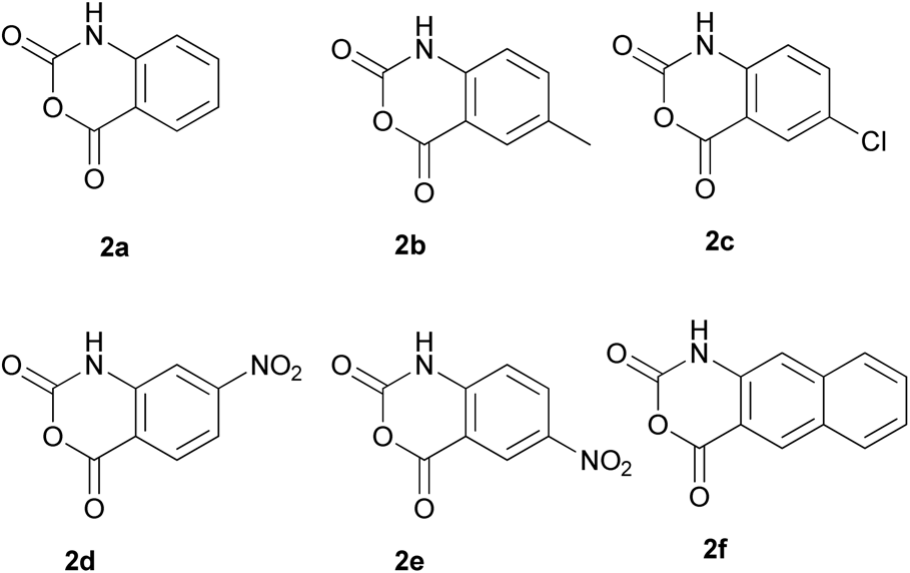
1*H*-Benzo[*d*][1,3]oxazine-2,4-dione derivatives synthesized by the proposed two-step procedure of Scheme 5.

Taking advantage of these findings, we propose a two-step approach for the synthesis of the desired 1*H*-benzo[*d*][1,3]oxazine-2,4-diones (2a–2f) starting from 2-aminobenzoic acids such as the 1a–1e or similar *o*-aminobenzoic acid substrates, like the 3-amino-2-naphtoic acid (1f) (Scheme 5). The proposed method involves the reaction of EtOCOCl (a cheap and easy-to-handle starting material, which allows easy transformation through the release of EtCl and 1*H*-benzo[*d*][1,3]oxazine-2,4-dione) with 1a–e; 1f in presence of Na_2_CO_3_/NaHCO_3_ in water/acetone (1 : 4) (a method that offers scalability potential^31^ and makes use of acetone – a common and widely used solvent in organic chemistry) to initially afford the EtOCO derivatives. These are then treated with 5-fold molar excess of SOCl_2_ in THF at room temperature (as the use of a 2-fold molar excess was found to result in incomplete transformations), without being previously isolated, to finally afford the desired 1*H*-benzo[*d*][1,3]oxazine-2,4-diones 2a–e and 1*H*-naphtho[2,3-*d*][1,3]oxazine-2,4-dione (2f) (Fig. 2). The total yields (over 2 steps) ranged from 65 to 80% in all cases tested, while the NMR analysis (Fig. S17–S22†) and HPLC analysis (Fig. S23†) revealed the high purity of the obtained products.

### 1*H*-Benzo[*d*][1,3]oxazine-2,4-diones from RCH_2_OCO-2-aminobenzoic acids – mechanistic aspects

Although the general cyclization mechanism for these transformations seems rather self-evident, proceeding through the initial carboxylic acid activation which enables cyclization to 2-alkyloxy-4*H*-3,1-benzo[*d*][1,3]oxazin-4-one and nucleophilic attack of chloride anions on the methylene group of 2-alkyloxy-4*H*-3,1-benzo[*d*][1,3]oxazin-4-one (Scheme 2), we were not able to identify this intermediate in the NMR spectra.

Thus, in order to provide further mechanistic insights, we synthesized 2-ethoxy-4*H*-benzo[*d*][1,3]oxazin-4-one.^32^ The NMR of this product is provided in the ESI file (Fig. S24A)† confirming its high purity. As can be seen from the HPLC analysis (Fig. S24B†), 2-ethoxy-4*H*-benzo[*d*][1,3]oxazin-4-one is partly hydrolyzed in the HPLC column to the corresponding EtOCO-2-aminobenzoic acid 7a. This information could safely indicate that the marked with asterisk peaks in the HPLC analysis during the treatment of Fmoc- and EtOCO-2-aminobenzoic acids with SOCl_2_ belong to the corresponding 2-alkyloxy-4*H*-benzo[*d*][1,3]oxazin-4-ones, partly hydrolyzed to the corresponding Fmoc/EtOCO-2-aminobenzoic acids during the HPLC analysis (Fig. S1, S2 and S7–S10†).

**Scheme 6:**
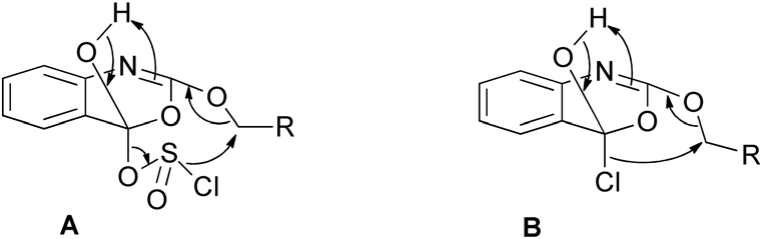
Possible mechanistic approaches for the chlorination of the methylene group of the urethane type groups of 2-aminobenzoic acids and subsequent release of 1*H*-benzo[*d*][1,3] oxazine-2,4-diones.

A possible explanation for the absence of 2-alkyloxy-4*H*-benzo[*d*][1,3]oxazin-4-one from the NMR data obtained during the treatment of EtOCO-2-aminobenzoic acid 7a with SOCl_2_ to the corresponding 2a (and Cbz-2-aminobenzoic 5a acid to 2a) could be the fast intramolecular nucleophilic attack of chloride to the adjacent methlylene group, once the cyclized ring is formed (by an initial nucleophilic attack of the neighboring carbamate oxygen to the activated – either as an acyl chloride or as a chlorosulfite – carboxylic acid group), as presented in Scheme 6. Although Scheme 6 only shows possible mechanistic pathways and further evidence would be required to support them, these could explain the absence of any 2-alkyloxy-4*H*-benzo[*d*][1,3]oxazin-4-ones from the NMR spectra during transformation and the selectivity of the reaction to finally afford 2 and RCH_2_Cl.

To further support our findings and highlight the significance of the proposed method we also performed the reaction of 2-ethoxy-4*H*-benzo[*d*][1,3]oxazin-4-one with LiCl and LiBr under identical conditions (LiX (10 eq.) in THF (0.2 M) at room temperature). The reactions were performed in the absence and presence of an acid (1% & 10% AcOH). Although the presence of an acid seems to improve the reaction rate (especially in the case of LiBr), what is of particular importance in this study is that the reaction rates are low (in all cases tested), while selectivity issues seem to arise that determine the reaction progress, leading to incomplete transformations to the desired 2a (Fig. S25–S28†). In contrast, the treatment of 7a with a 5-fold molar excess of SOCl_2_ results in the formation of only 2a within 7–8 h at room temperature.

## Conclusions

An investigation for the synthesis of 1*H*-benzo[*d*][1,3]oxazine-2,4-diones through the reaction of SOCl_2_, a widely used solvent in pharmaceutical synthesis,^33–36^ with Fmoc-, Cbz- and EtOCO-2-aminobenzoic acids was conducted. Our investigations showed that the use of SOCl_2_ enables activation/cyclization and final transformation at room temperature, proceeding through the nucleophilic attack of chloride anions to the methylene group of 2-alkyloxy-4*H*-3,1-benzo[*d*][1,3]oxazin-4-one, to afford the expected 1*H*-benzo[*d*][1,3]oxazine-2,4-diones and RCH_2_Cl (CMF, benzyl chloride, and ethyl chloride). For this, a 5-fold molar excess of SOCl_2_ is needed to drive the transformation into completion (in the case of Fmoc-2-aminobenzoic acids a 10-fold molar excess is needed). Taking advantage of our initial findings, we rationalized the use of EtOCOCl, a cheap and easy-to-handle starting material, as the source of the carbonyloxy group, and we finally propose a two-step procedure for the synthesis of 1*H*-benzo[*d*][1,3]oxazine-2,4-diones starting from 2-aminobenzoic acids, to initially form ethoxycarbonyl-2-aminobenzoic acids, subsequently treated with a 5 molar excess of SOCl_2_. Significantly, the proposed protocol does not require the isolation of the initially formed ethoxycarbonyl-2-aminobenzoic acids, while the cyclization and products formation is easily achieved – at room temperature – without any unwanted reactions/by-products. Thus, a valuable method is proposed for the transformation of 2-aminobenzoic acids to 1*H*-benzo[*d*][1,3]oxazine-2,4-diones, using cheap and easy-to-handle materials, reduced amounts of solvents, waste, and preparation time, yet with high yields and purities.

## Experimental

### Materials

Anthranilic acid (2-aminobenzoic acid) (1a) was purchased from Thermo Scientific Chemicals (Acros) (Geel, Belgium). 2-Aminobenzoic acids 1b–e and 3-amino-2-naphtoic acid 1f were purchased from BLD Pharmatech GmbH (Reinbek, Germany). Tetrahydrofuran ACS, Reag. Ph. Eur. ≥99.9% (containing BTH as stabilizer) was purchased from Honeywell (Seetze, Germany). Diethylether ACS, Reag. USP, Ph. Eur. ≥99.7% (containing BTH as stabilizer) was purchased from AppliChem GmbH (Darmstadt, Germany). MeOH for HPLC were purchased from Fischer Scientific (Geel, Belgium). Acetonitrile and water for HPLC were kindly provided by CBL Patras S.A. (Patras, Greece). All other chemicals were purchased from Sigma-Aldrich (Darmstadt, Germany) and Merck (Darmstadt, Germany). A freshly opened bottle of thionyl chloride was used throughout this work.

### Analytical methods

Thin layer chromatography (TLC) was performed on silica gel 60 F254 plates (Merck, Darmstadt, Germany) and spot detection was carried out by UV light. HPLC analysis was performed on a Waters 2695 multisolvent delivery system (Milford, MA, USA), combined with a Waters 991 photodiode array detector, using a Puroshpere RP-8 (5 μm); 250 mm to 4 mm, and a Lichrosphere RP-8e (5 μm); 125 mm to 4 mm HPLC column; ESI-MS spectra were recorded on a Micromass Platform L.C. (Manchester, UK) at 30 V. NMR spectra were obtained at 600 MHz, on a Bruker Avance III HD spectrometer at 298.1 K. Chemical shifts (*δ*) were referenced to the corresponding solvent peaks and are reported in ppm.

## Experimental procedures

### Synthesis of 3a, 3b, 5a, 5b, 7a

#### Fmoc-2-aminobenzoic acid 3a

2-Aminobenzoic acid (0.0146 mol; 2 g) was placed in a round-bottom flask and the solid was dissolved in a mixture of dioxane and aq. 10% Na_2_CO_3_ (1 : 1) (40 mL). The mixture was kept under stirring at room temperature, and *N*-(9-fluorenylmethoxycarbonyloxy) succinimide (Fmoc-OSu) (0.016 mol; 5.41 g) dissolved in 20 mL dioxane was slowly added and the pH of the reaction was periodically adjusted to around 8.0–9.0 (for the next 4–5 h) using aq. 10% Na_2_CO_3_, and the reaction mixture was further stirred overnight at room temperature. Then, ethyl acetate (EtOAc) was added (40 mL) and to this mixture conc. HCl was slowly added until pH 2.0. The two phases were separated, and the aqueous phase was washed twice with EtOAc. The combined organic phases were washed with water (2 × 50 mL) and then dried with Na_2_SO_4_, filtered and the filtering was concentrated into a rotary evaporator where a white solid was formed. This was delivered by washings with diethyl ether (DEE) (3 × 50 mL) and then dried *in vacuo*. Yield: Fmoc-2-aminobenzoic acid 3a: 64%. ^1^H-NMR *δ* (600 MHz, DMSO-*d*_6_) *δ* 13.70 (s), 10.81 (s), 8.15 (d, *J* = 5.6 Hz), 7.98 (d, *J* = 7.7 Hz), 7.92 (d, *J* = 7.4 Hz), 7.69 (d, *J* = 7.3 Hz), 7.57 (t, *J* = 7.5 Hz), 7.43 (t, *J* = 7.5 Hz), 7.35 (t, *J* = 7.4 Hz), 7.11 (t, *J* = 7.5 Hz), 4.49 (d, *J* = 6.7 Hz), 4.36 (t, *J* = 6.6 Hz); ^13^C NMR *δ* (151 MHz, DMSO-*d*_6_) *δ* 170.57, 153.59, 144.52, 141.73, 141.70, 135.14, 132.11, 128.63, 128.05, 125.88, 122.93, 121.10, 119.36, 116.74, 67.17, 47.39. ESI-MS (calculated for C_22_H_17_NO_4_; exact mass (M + H): 360.12; found: 360.89).

#### Fmoc-2-amino-5-methylbenzoic acid 3b

2-Amino-5-methylbenzoic acid (0.132 mol; 2 g) and Fmoc-OSu (0.0146 mol; 4.91 g) were reacted by the previously reported method (for 3a synthesis). Yield: Fmoc-2-aminobenzoic acid 3b: 64%; Fmoc-2-amino-5methylbenzoic acid 3b: 62%. ^1^H NMR *δ* (600 MHz, DMSO-*d*_6_) *δ* 13.62 (s, 1H), 10.65 (s, 1H), 8.00 (d, *J* = 49.3 Hz, 1H), 7.91 (d, *J* = 7.5 Hz, 2H), 7.78 (d, *J* = 1.4 Hz, 1H), 7.68 (d, *J* = 7.5 Hz, 2H), 7.43 (t, *J* = 7.4 Hz, 2H), 7.39 (d, *J* = 8.0 Hz, 1H), 7.34 (td, *J* = 7.4, 0.8 Hz, 2H), 4.48 (d, *J* = 6.9 Hz, 2H), 4.35 (t, *J* = 6.8 Hz, 1H), 2.28 (s, 3H); ^13^C NMR *δ* (151 MHz, DMSO-*d*_6_) *δ* 170.63, 153.64, 144.58, 141.72, 139.35, 135.77, 132.12, 132.02, 128.66, 128.08, 125.93, 121.15, 119.48, 116.70, 67.12, 47.41, 20.99. ESI-MS (calculated for C_23_H_19_NO_4_; exact mass (M + H): 374.14; found: 373.99).

#### Cbz-2-aminobenzoic acid 5a

2-Aminobenzoic acid (7.29 mmol; 1 g) was placed in a round-bottom flask and the solid was dissolved in 30 mL H_2_O, Na_2_CO_3_ (1.55 g; 14.58 mmol) and NaHCO_3_ (0.61 g; 7.29 mmol), and then acetone (120 mL) was added. The mixture was placed in an ice-water bath (around 10 °C) and benzyl chloroformate (9.115 mmol; 1.30 mL) was slowly added (within 30–45 min), and the reaction mixture was further stirred for 3 h (at around 10 °C) and then overnight (at room temperature). Then, acetone was evaporated, and 20 mL H_2_O were added to dissolve the solid that was formed. The pH of the aqueous phase was adjusted to 8.0–9.0 (if needed) and then washed with DEE (3 × 20 mL). To the resulting aqueous phase, ethyl EtOAc was added (30 mL) and the aqueous phase was acidified by conc. HCl until pH 2.0. The organic phase was further washed with water (2 × 30 mL) and then dried with Na_2_SO_4_, filtered and the filtering was concentrated into a rotary evaporator where a white solid was formed. This was obtained by washings with hexane (Hex) (3 × 30 mL) and then dried *in vacuo*. Yield: Cbz-2-aminobenzoic acid 5a: 60%. ^1^H NMR (600 MHz, CDCl_3_) *δ* 10.28 (s, 1H), 8.48 (d, *J* = 8.5 Hz, 1H), 8.09 (dd, *J* = 8.0, 1.4 Hz, 1H), 7.61–7.55 (m, 1H), 7.43 (d, *J* = 7.3 Hz, 2H), 7.38 (t, *J* = 7.4 Hz, 2H), 7.33 (t, *J* = 7.2 Hz, 1H), 7.09–7.03 (m, 1H), 5.23 (s, 2H); ^13^C NMR *δ* (151 MHz, CDCl_3_) *δ* 172.84, 153.40, 142.32, 136.04, 135.76, 131.97, 128.61, 128.39, 128.36, 121.90, 119.08, 113.49, 67.09. ESI-MS (calculated for C_15_H_13_NO_4_; exact mass (M + H): 272.09; found: 272.12).

#### Cbz-2-amino-5-methylbenzoic acid 5b

2-Amino-5-methylbenzoic acid (6.62 mmol; 1 g) was placed in a round-bottom flask and the solid was dissolved in 30 mL H_2_O, Na_2_CO_3_ (1.40 g; 13.23 mmol) and NaHCO_3_ (0.56 g; 6.62 mmol), and then acetone (120 mL) was added. The mixture was placed in an ice-water bath (around 10 °C) and benzyl chloroformate (8.27 mmol; 1.18 mL) was slowly added (within 30–45 min), and the reaction mixture was further stirred for 3 h (at around 10 °C) and then overnight (at room temperature). Then, acetone was evaporated, and 20 mL H_2_O were added to dissolve the solid that was formed, and the aqueous phase was washed with DEE (3 × 20 mL). To the resulting aqueous phase, EtOAc was added (30 mL) and the aqueous phase was acidified by conc. HCl until pH 2.0. The organic phase was dried with Na_2_SO_4_, filtered and the filtering was concentrated into a rotary evaporator where a white solid was formed. This was obtained by washings with hexane (Hex) (3 × 50 mL) and then dried *in vacuo*. Yield: Cbz-2-amino-5-methylbenzoic acid 5b: 65%. ^1^H NMR (600 MHz, CD_3_OD) *δ* 8.24 (d, *J* = 8.4 Hz, 1H), 7.86 (s, 1H), 7.49–7.20 (m, 6H), 5.19 (s, 2H), 2.31 (s, 3H); ^13^C NMR (151 MHz, CD_3_OD) *δ* 172.22, 155.80, 141.31, 138.73, 136.85, 133.47, 133.42, 130.43, 130.08, 129.97, 120.54, 117.33, 68.65, 21.41. ESI-MS (calculated for C_16_H_15_NO_4_; exact mass (M + H): 286.11; found: 286.14).

#### EtOCO-2-aminobenzoic acid 7a

2-Aminobenzoic acid (7.29 mmol; 1 g) was placed in a round-bottom flask and the solid was dissolved in 30 mL H_2_O, Na_2_CO_3_ (1.55 g; 14.58 mmol) and NaHCO_3_ (0.61 g; 7.29 mmol), and then acetone (120 mL) was added. The mixture was placed in an ice-water bath around 10–15 °C and ethyl chloroformate (9.115 mmol; 0.87 mL) was slowly added (within 30–45 min), and the mixture was stirred overnight. Then, acetone was evaporated, and 30 mL H_2_O were added to dissolve the solid that was formed. The pH of the aqueous phase was adjusted to 8.0–9.0 (if needed) and then washed with DEE (3 × 20 mL). To the resulting aqueous phase, EtOAc was added (30 mL) and the aqueous phase was acidified by HCl until pH 2. The organic phase was dried with Na_2_SO_4_, filtered and the filtering was concentrated into a rotary evaporator. The solid (or oily) product that was formed was further treated with DEE and the solid that was formed was filtered and washed with DEE (3 × 20 mL) and dried *in vacuo*. Yield: EtOCO-2-aminobenzoic acid 7a: 60%. ^1^H NMR (600 MHz, DMSO-*d*_6_) *δ* 13.48 (s, 1H), 8.13 (d, *J* = 8.1 Hz, 1H), 7.96 (d, *J* = 7.3 Hz, 1H), 7.27 (t, *J* = 7.3 Hz, 1H), 6.89 (t, *J* = 7.3 Hz, 1H), 4.09 (dd, *J* = 14.0, 7.0 Hz, 2H), 1.22 (t, *J* = 7.0 Hz, 3H); ^13^C NMR (151 MHz, DMSO-*d*_6_) *δ* 171.48, 154.19, 141.68, 132.24, 131.30, 124.43, 121.31, 117.63, 60.74, 15.50. ESI-MS (calculated for C_10_H_11_NO_4_; exact mass (M + H): 210.08; found: 210.11).

#### Synthesis of 2a–f from 1a–f

The following quantities of starting materials and reagents were used: 1a: 1 g, 7.29 mmol; Na_2_CO_3_: 1.5 g, 14.58 mmol; NaHCO_3_: (0.61 g, 7.29 mmol), EtOCOCl (9.11 mmol, 0.87 mL); 1b: 1 g, 6.62 mmol; Na_2_CO_3_: 1.40 g, 13.23 mmol; NaHCO_3_: 0.56 g, 6.62 mmol), EtOCOCl (8.27 mmol, 0.79 mL); 1c: 1 g, 5.83 mmol; Na_2_CO_3_: 1.24 g, 11.66 mmol; NaHCO_3_: 0.49 g, 5.83 mmol), EtOCOCl (7.29 mmol, 0.69 mL); 1d: 1 g, 5.49 mmol; Na_2_CO_3_: 1.16 g, 10.98 mmol; NaHCO_3_: 0.46 g, 5.49 mmol), EtOCOCl (6.86 mmol, 0.65 mL); 1e: 1 g, 5.49 mmol; Na_2_CO_3_: 1.16 g, 10.98 mmol; NaHCO_3_: 0.46 g, 5.49 mmol), EtOCOCl (6.86 mmol, 0.65 mL); 1f: 1 g, 5.34 mmol; Na_2_CO_3_: 1.13 g, 10.68 mmol; NaHCO_3_: 0.45 g, 5.34 mmol), EtOCOCl (6.68 mmol, 0.64 mL).

The general procedure is as follows: 2-aminobenzoic acid (1a–e); 3-amino-2-naphtoic acid 1f was placed in a round-bottom flask and the solid was dissolved in 30 mL H_2_O, Na_2_CO_3_ and NaHCO_3_, and then acetone (120 mL) was added. The mixture was placed in an ice water bath (around 10 °C) and ethyl chloroformate was slowly added (within 30–45 min), and the reaction mixture was further stirred for 3 h (at around 10 °C) and then overnight (at room temperature). Then, acetone was evaporated, and 20 mL H_2_O was added to dissolve the solid that was formed. The pH of the aqueous phase was adjusted to 8.0–9.0 (if needed) and then washed with diethyl ether (DEE) (3 × 20 mL). To the resulting aqueous phase, ethyl acetate (EtOAc) was added (30 mL) and the aqueous phase was acidified with concentrated HCl until pH 2.0. The organic phase was further washed with water (2 × 30 mL) and then dried with Na_2_SO_4_, filtered and the filtering was concentrated. The solid (or oily) product that was formed was directly dissolved in THF (0.2 M corresponding to the moles of 1a–f) and to the resulting solution, SOCl_2_ (5 eq. corresponding to the moles of 1a–f) was added and the mixture was stirred at room temperature, overnight. Then, the reaction mixture was concentrated, and the solid that was formed was filtered and washed with DEE (3 × 30 mL) and dried to afford 2a–f. Total yields (over 2 steps) 65–80%.

2a (1*H*-Benzo[*d*][1,3]oxazine-2,4-dione): yield 67%; ^1^H NMR (600 MHz, DMSO-*d*_6_) *δ* 11.72 (s, 1H), 7.92 (dd, *J* = 7.9, 1.0 Hz, 1H), 7.77–7.71 (m, 1H), 7.28–7.22 (m, 1H), 7.15 (d, *J* = 8.2 Hz, 1H); ^13^C NMR (151 MHz, DMSO-*d*_6_) *δ* 160.81, 148.02, 142.33, 137.86, 129.86, 124.44, 116.26, 116.23, 111.20. ESI-MS (calculated for C_8_H_5_NO_3_; exact mass (M + H): 164.03; found: 164.09).

2b (6-Methyl-1*H*-benzo[*d*][1,3]oxazine-2,4-dione): yield 72%; ^1^H NMR (600 MHz, DMSO-*d*_6_) *δ* 11.64 (s, 1H), 7.72 (s, 1H), 7.57 (dd, *J* = 8.3, 1.5 Hz, 1H), 7.06 (d, *J* = 8.3 Hz, 1H), 2.33 (s, 3H); ^13^C NMR (151 MHz, DMSO-*d*_6_) *δ* 160.83, 148.04, 140.16, 138.86, 133.86, 129.24, 116.20, 110.93, 20.98. ESI-MS (calculated for C_9_H_7_NO_3_; exact mass (M + H): 178.05; found: 178.10).

2c (6-Chloro-1*H*-benzo[*d*][1,3]oxazine-2,4-dione): yield 72%; ^1^H NMR (600 MHz, DMSO-*d*_6_) *δ* 11.86 (s, 1H), 7.86 (d, *J* = 2.3 Hz, 1H), 7.77 (dd, *J* = 8.7, 2.4 Hz, 1H), 7.16 (d, *J* = 8.7 Hz, 1H); ^13^C NMR (151 MHz, DMSO-*d*_6_) *δ* 159.97, 147.78, 141.28, 137.66, 128.68, 128.18, 118.47, 113.01. ESI-MS (calculated for C_8_H_4_ClNO_3_; exact mass (M + H): 198.00; found: 198.12).

2d (7-Nitro-1*H*-benzo[*d*][1,3]oxazine-2,4-dione): yield 80%; ^1^H NMR (600 MHz, DMSO-*d*_6_) *δ* 12.09 (s, 1H), 8.14 (t, *J* = 10.5 Hz, 1H), 7.96 (dd, *J* = 8.6, 2.0 Hz, 1H), 7.86 (d, *J* = 2.0 Hz, 1H); ^13^C NMR (151 MHz, DMSO-*d*_6_) *δ* 159.71, 152.88, 147.56, 143.02, 131.85, 118.24, 116.40, 111.15. ESI-MS (calculated for C_8_H_4_N_2_O_5_; exact mass (M + H): 209.02; found: 209.10).

2e (6-Nitro-1*H*-benzo[*d*][1,3]oxazine-2,4-dione): yield 74%; ^1^H NMR (600 MHz, DMSO-*d*_6_) *δ* 12.36 (s, 1H), 8.56 (dd, *J* = 10.7, 4.3 Hz, 1H), 8.52 (dd, *J* = 9.0, 2.6 Hz, 1H), 7.33 (t, *J* = 11.8 Hz, 1H); ^13^C NMR (151 MHz, DMSO-*d*_6_) *δ* 159.56, 147.43, 146.99, 143.36, 132.17, 125.47, 117.60, 112.05. ESI-MS (calculated for C_8_H_4_N_2_O_5_; exact mass (M + H): 209.02; found: 209.12).

2f (1*H*-Naphtho[2,3-*d*][1,3]oxazine-2,4-dione): yield 70%; ^1^H NMR (600 MHz, DMSO-*d*_6_) *δ* 11.77 (s, 1H), 8.69 (d, *J* = 16.0 Hz, 1H), 8.11 (d, *J* = 8.2 Hz, 1H), 7.92 (d, *J* = 8.3 Hz, 1H), 7.65 (dd, *J* = 16.6, 9.2 Hz, 1H), 7.52–7.44 (m, 2H); ^13^C NMR (151 MHz, DMSO-*d*_6_) *δ* 160.90, 147.86, 137.90, 136.91, 132.67, 131.11, 130.62, 129.80, 127.82, 126.41, 112.00, 111.33. ESI-MS (calculated for C_12_H_7_NO_3_; exact mass (M + H): 214.05; found: 214.11).

## Author contributions

Conceptualization, S. M.; data curation, S. M.; formal analysis, S. M., N. M., V. A.; funding acquisition/project administration, S. M.; investigation, S. M., N. M., V. A.; methodology, S. M.; supervision, S. M.; writing – original draft, S. M.; writing – review and editing, S. M., V. A. All authors have read and agreed to the published version of the manuscript.

## Conflicts of interest

There are no conflicts to declare.

## Supplementary Material

RA-015-D5RA04014K-s001

## Data Availability

The data supporting this article have been included as part of the ESI.†
