# Supplementary material for: Investigation for the easy and efficient synthesis of 1H-benzo[d][1,3]oxazine-2,4-diones
Source: RSC Adv. 2025 Aug 4;15(34):27644–51. doi: 10.1039/d5ra04014k (PMC12320480; doi:10.1039/d5ra04014k)
Supplement: RA-015-D5RA04014K-s001 [file RA-015-D5RA04014K-s001.pdf]

Supplementary Information (SI) for RSC Advances.
This journal is © The Royal Society of Chemistry 2025

# Investigation for the easy and efficient synthesis of 1*H*-benzo[*d*][1,3]oxazine-2,4-diones

Nikolaos Mitsostergios,[a] Vasileios Athanasopoulos [a] and Spyridon Mourtas[a,*]

[a] Department of Chemistry, University of Patras, 26510 Rio Patras, Greece

## Supporting Information

### A) Fmoc-2ABA-OH and Fmoc-2AMBA-OH + $SOCl_2$ study

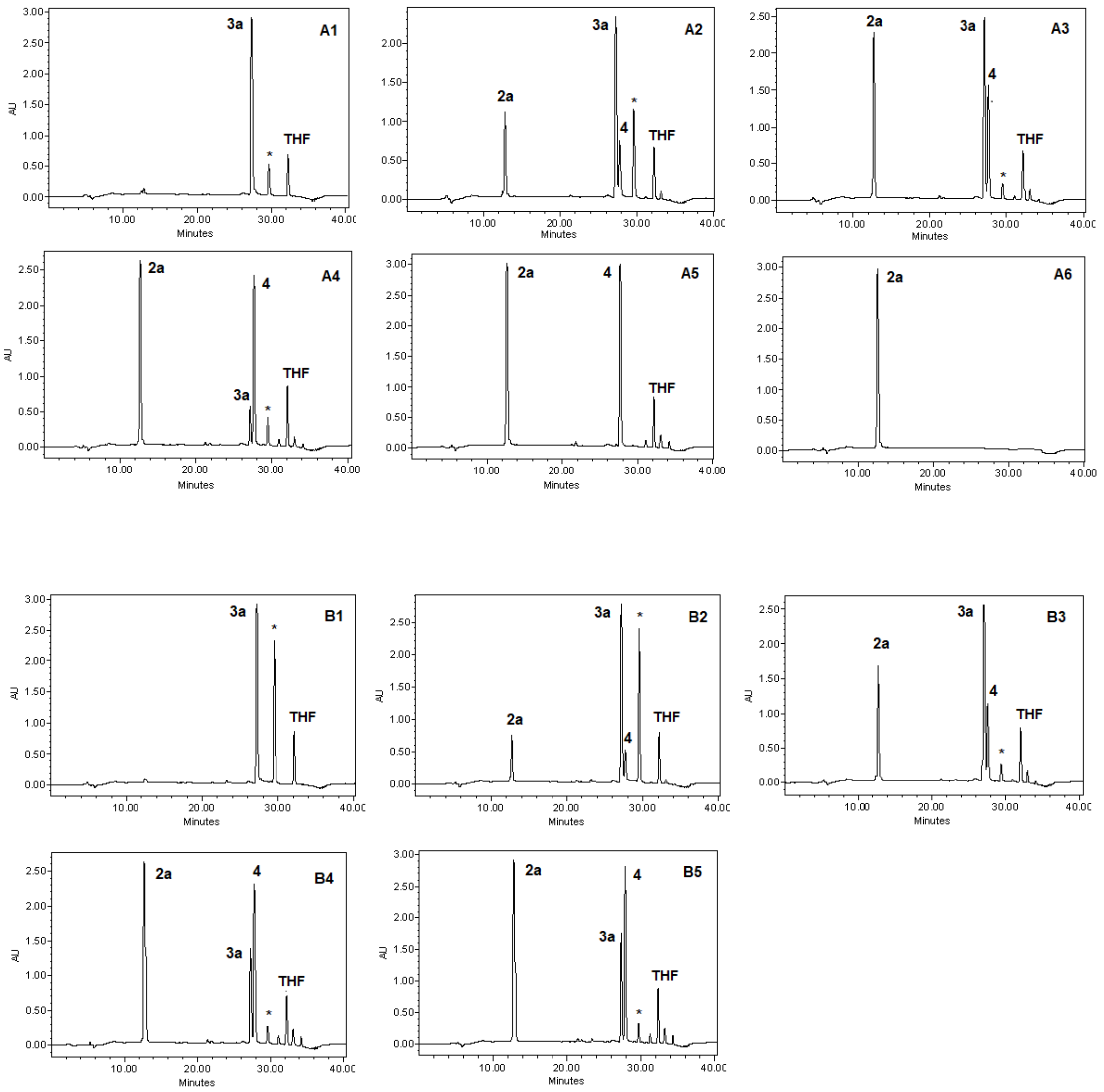


**Figure S1.** Representative hplc analysis for the conversion of **3a** to **2a** and **4** in presence of $SOCl_2$. Reaction conditions: 10 molar excess (**A1**–**A6**) of $SOCl_2$ in respect to **3a** in THF (containing BHT as stabilizer) (0.2 M) at room temperature (22–24 °C). Hplc profile during the reaction mixture at 2 h (**A1**), 12 h (**A2**), 24 h (**A3**), 2 d (**A4**), 3 d (**A4**), Isolated **2a** (**A6**); 5 molar excess (**B1**–**B5**) of $SOCl_2$ in respect to **3a** in THF (containing BHT as stabilizer) (0.2 M) at room temperature (22–24 °C). Hplc profile during the reaction mixture at 2 h (**B1**), 12 h (**B2**), 24 h (**B3**), 2 d (**B4**), 3 d (**B5**). Hplc column/conditions: Puroshpere RP-8 (5μm); 250mm–4mm; 0.5 ml/min; 20% to 100% AcCN in 30 min; Abs: 214 nm.

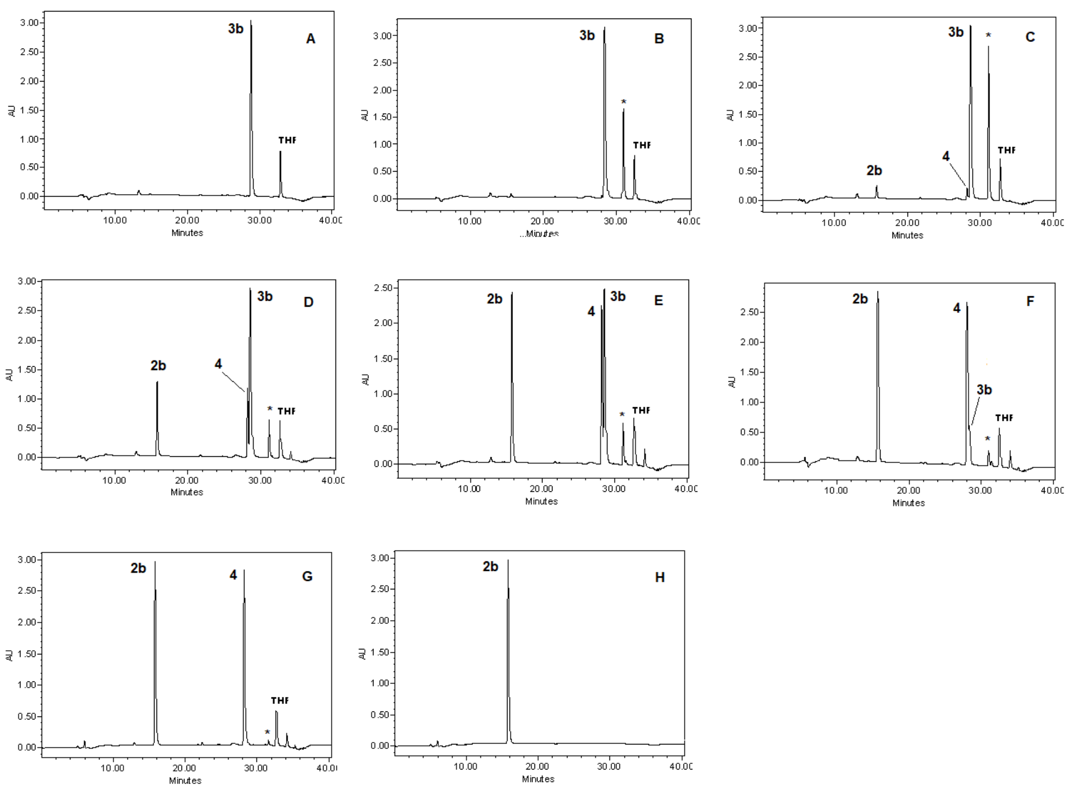


**Figure S2.** Representative hplc analysis for the conversion of **3b** to **2b** and **4** in presence of $SOCl_2$. Reaction conditions: 10 molar excess of $SOCl_2$ in respect to **3b** in THF (containing BHT as stabilizer) (0.2 M) at room temperature (22–24 $^{o}$C). Hplc profile during the reaction mixture at 30 min (**A**), 1 h (**B**), 2 h (**C**), 12 h (**D**), 24 h (**E**), 48 h (**F**), 3 d (**G**), Isolated **2b** (**H**). Hplc column/conditions: Puroshpere RP-8 (5μm); 250mm–4mm; 0.5 ml/min; 20% to 100% AcCN in 30 min; Abs: 214 nm.

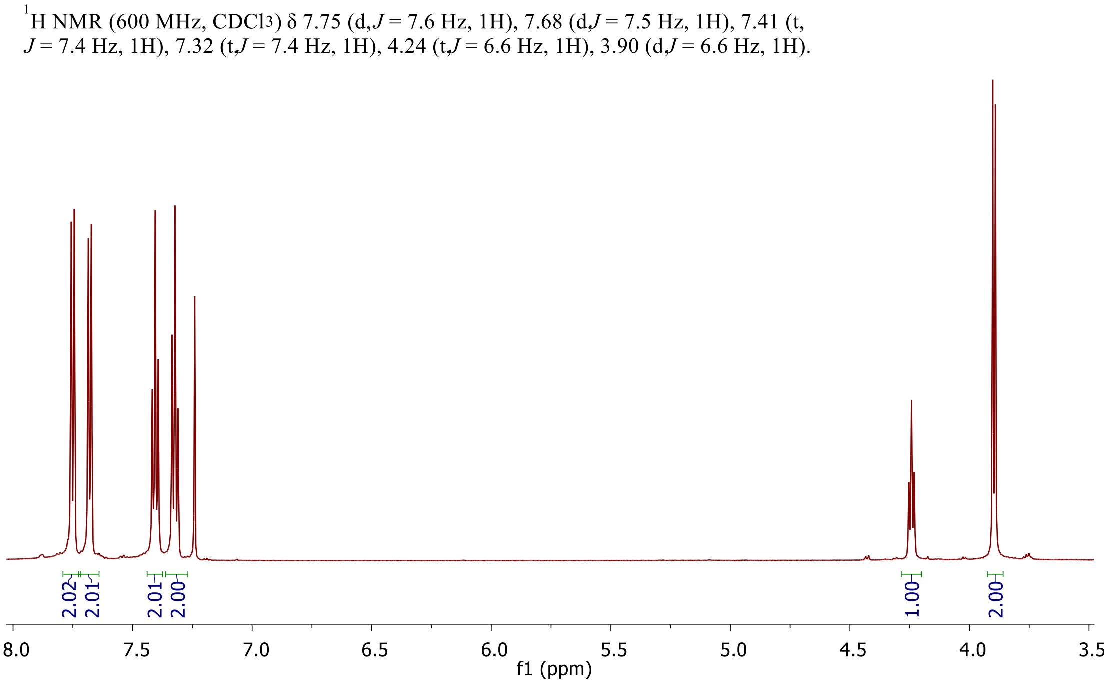


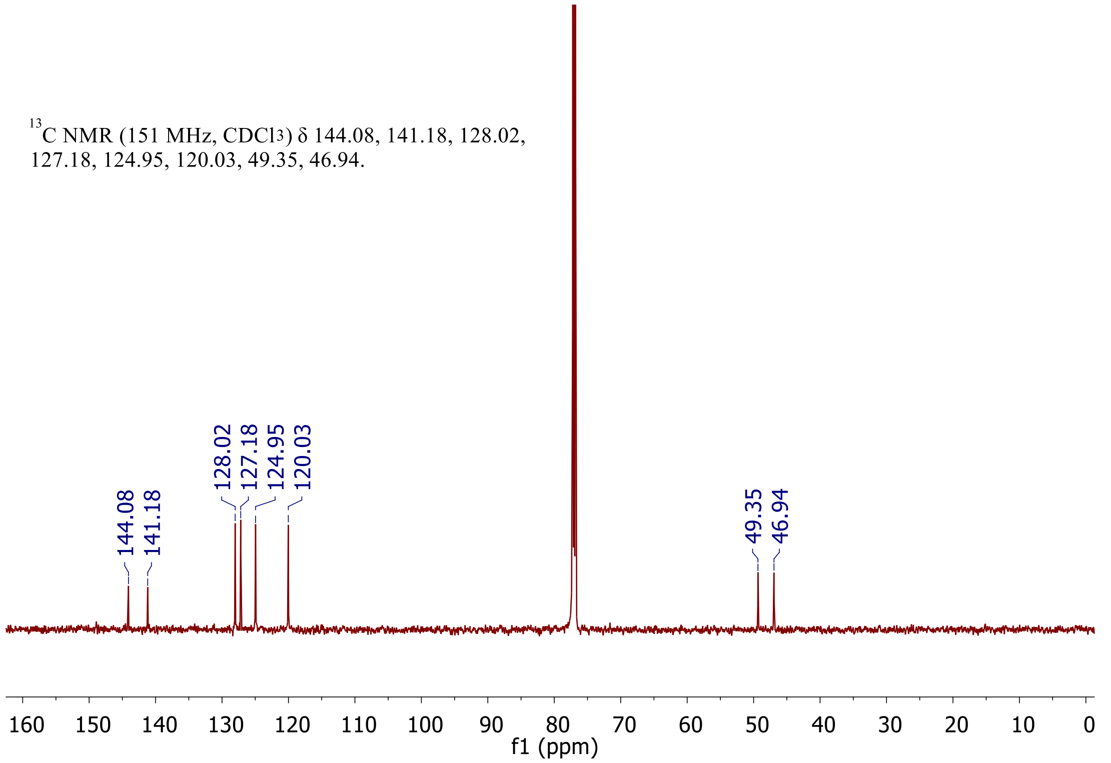


**Figure S3.** $^{1}$H and $^{13}$C-NMR of CMF (**4**) (collected by semi-preparative hplc and lyophilized). The same product formation (**4**) was identified for both **3a** (Figure S1) and **3b** (Figure S2) treatment with $SOCl_2$. The NMR recorded for CMF (**4**) is in accordance with previously published data for this product.[35] ; $^{1}$H NMR (600 MHz, $CDCl_3$) *δ* 7.75 (d, *J* = 7.6 Hz, 1H), 7.68 (d, *J* = 7.5 Hz, 1H), 7.41 (t, *J* = 7.4 Hz, 1H), 7.32 (t, *J* = 7.4 Hz, 1H), 4.24 (t, *J* = 6.6 Hz, 1H), 3.90 (d, *J* = 6.6 Hz, 1H); $^{13}$C NMR (151 MHz, $CDCl_3$) *δ* 144.08, 141.18, 128.02, 127.18, 124.95, 120.03, 49.35, 46.94.

## B) Cbz-2ABA-OH and Cbz-2AMBA+ $SOCl_2$ study

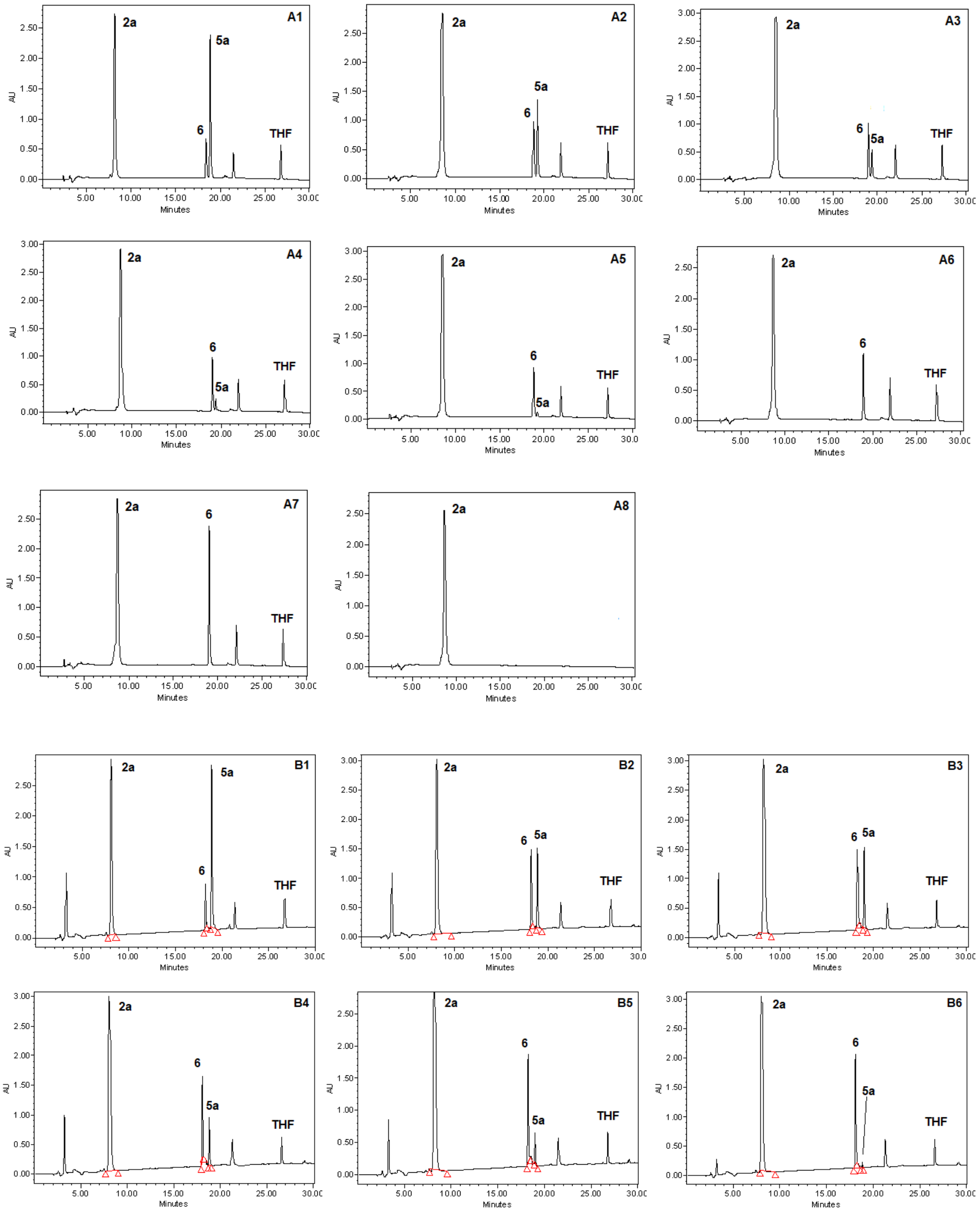

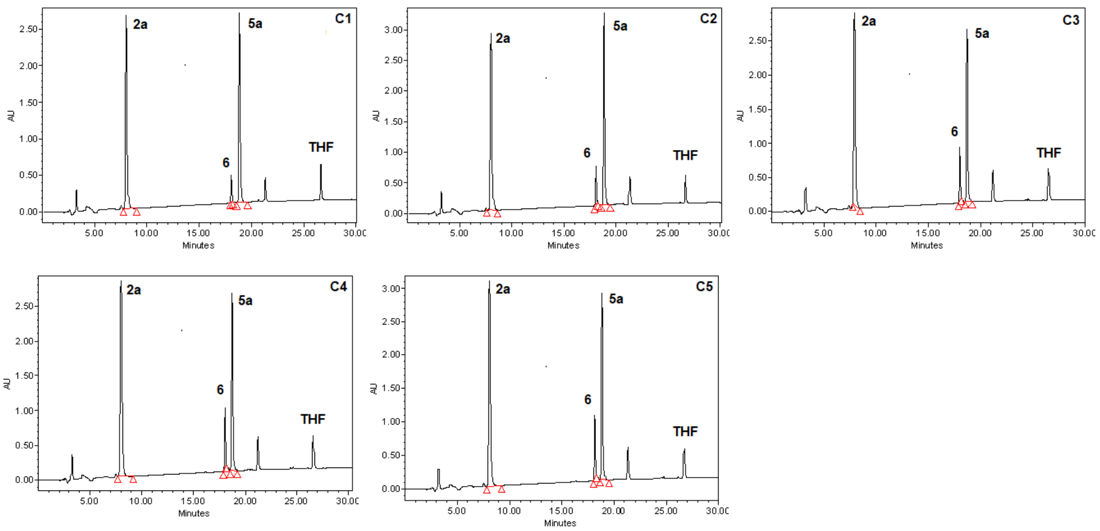


**Figure S4.** Representative hplc analysis for the conversion of **5a** to **2a** and **6** in presence of $SOCl_2$. Reaction conditions: 10 molar excess (**A1**–**A8**) of $SOCl_2$ in respect to **5a** in THF (containing BHT as stabilizer) (0.2 M) at room temperature (22–24 $^{o}$C). Hplc profile during the reaction mixture at 1 h (**A1**), 2 h (**A2**), 3 h (**A3**), 4 h (**A4**), 5 h (**A5**), 12 h (**A6**), 12 h + spike with Bz-Cl (**6**) (**A7**), Isolated **2a** (**A8**); 5 molar excess (**B1**–**B6**) of $SOCl_2$ in respect to **5a** in THF (containing BHT as stabilizer) (0.2 M) at room temperature (22–24 $^{o}$C). Hplc profile during the reaction mixture at 1 h (**B1**), 3 h (**B2**), 5 h (**B3**), 7 h (**B4**), 9 h (**B5**), 12 h (**B6**); 2 molar excess (**C1**–**C5**) of $SOCl_2$ in respect to **5a** in THF (containing BHT as stabilizer) (0.2 M) at room temperature (22–24 $^{o}$C). Hplc profile during the reaction mixture at 1 h (**C1**), 3 h (**C2**), 6 h (**C3**), 9 h (**C4**), 12 h (**C5**); Hplc column/conditions: Puroshpere RP-8 (5µm); 250mm–4mm; 1.0 ml/min; 20% to 100% AcCN in 30 min; Abs: 214 nm.

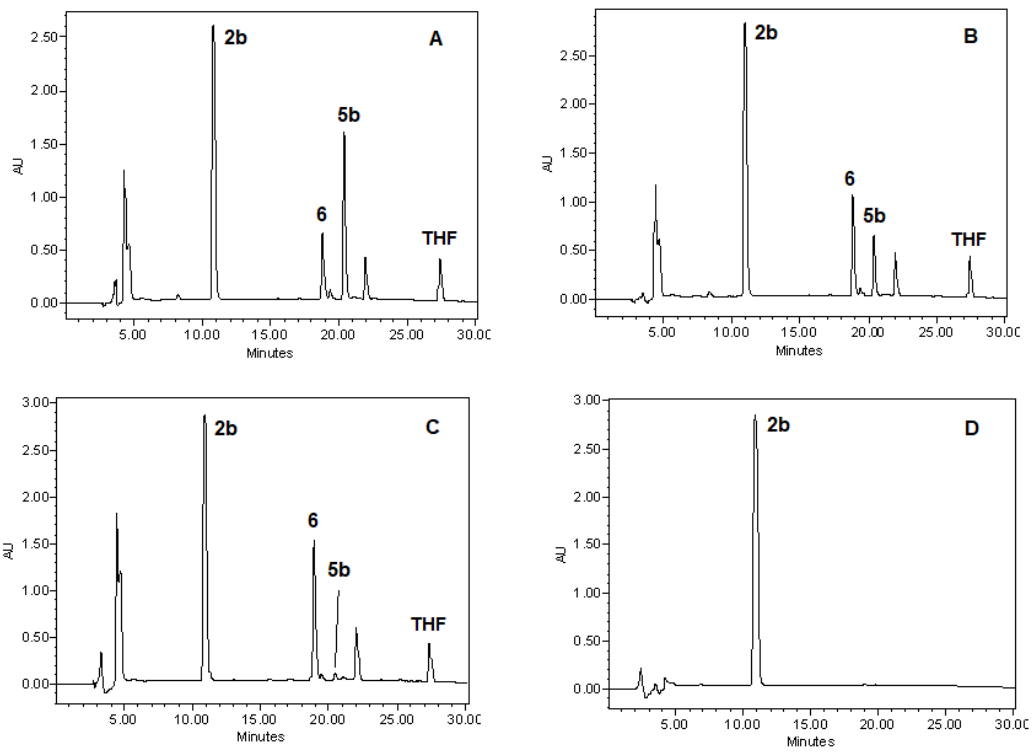


**Figure S5.** Representative hplc analysis for the conversion of **5b** to **2b** and **6** in presence of $SOCl_2$. Reaction conditions: 10 molar excess of $SOCl_2$ in respect to **5b** in THF (containing BHT as stabilizer) (0.2 M) at room temperature (22–24 $^{o}$C). Hplc profile during the reaction mixture at 1 h (**A**), 2 h (**B**), 4 h (**C**), Isolated **2b** (**D**). Hplc column/conditions: Puroshpere RP-8 (5μm); 250mm–4mm; 1.0 ml/min; 20% to 100% AcCN in 30 min; Abs: 214 nm.

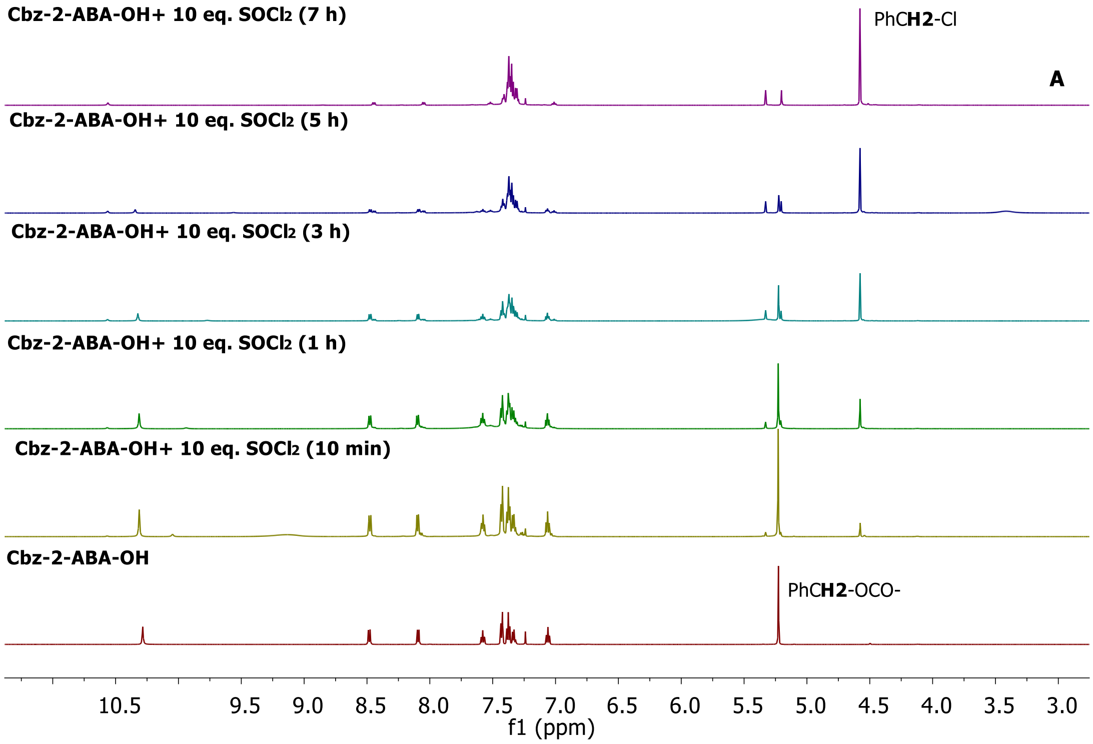

Cbz-2-ABA-OH+ 10 eq. SOCl2 (7 h)
PhCH2-Cl
A
Cbz-2-ABA-OH+ 10 eq. SOCl2 (5 h)
Cbz-2-ABA-OH+ 10 eq. SOCl2 (3 h)
Cbz-2-ABA-OH+ 10 eq. SOCl2 (1 h)
Cbz-2-ABA-OH+ 10 eq. SOCl2 (10 min)
Cbz-2-ABA-OH
PhCH2-OCO-
10.5 9.5 9.0 8.5 8.0 7.5 7.0 6.5 6.0 5.5 5.0 4.5 4.0 3.5 3.0
f1 (ppm)


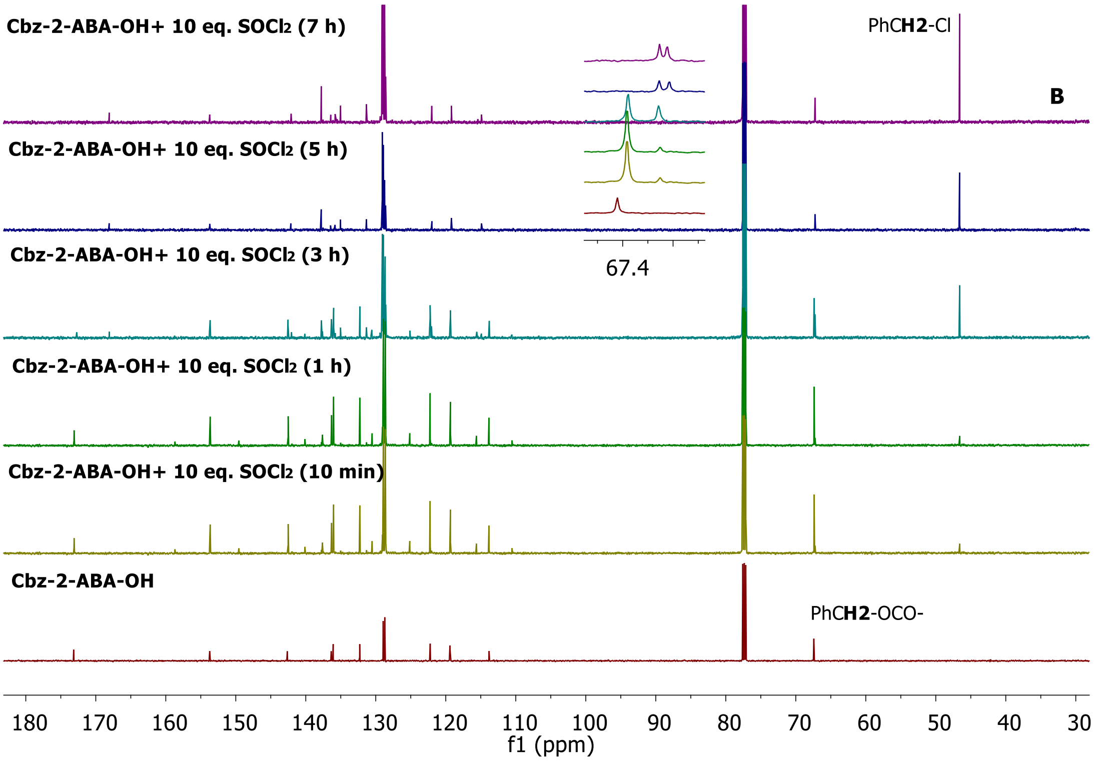

Cbz-2-ABA-OH+ 10 eq. SOCl2 (7 h)
PhCH2-Cl
B
Cbz-2-ABA-OH+ 10 eq. SOCl2 (5 h)
67.4
Cbz-2-ABA-OH+ 10 eq. SOCl2 (3 h)
Cbz-2-ABA-OH+ 10 eq. SOCl2 (1 h)
Cbz-2-ABA-OH+ 10 eq. SOCl2 (10 min)
Cbz-2-ABA-OH
PhCH2-OCO-
180 170 160 150 140 130 120 110 100 90 80 70 60 50 40 30
f1 (ppm)

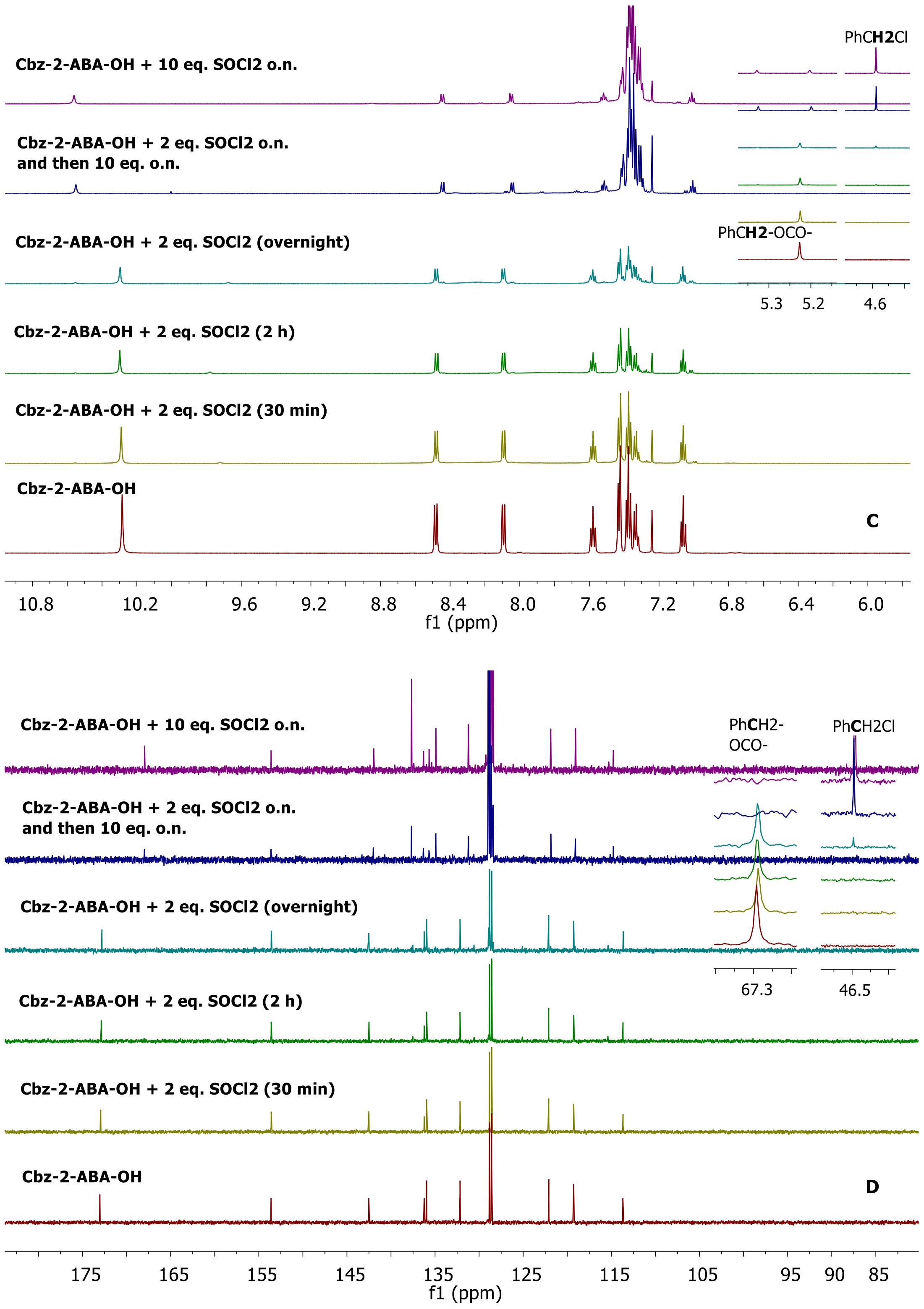


**Figure S6.** $^{1}$H-NMR and $^{13}$C-NMR of the reaction mixture of **5a** with $SOCl_2$, showing the formation of BzCl as the reaction released product. Reaction conditions: 10 molar excess of $SOCl_2$ in respect to **5a** in $CDCl_3$ (0.2 M) at room temperature (19–20 °C) (**A** & **B**); 2 molar excess of $SOCl_2$ in respect to **5a** in $CDCl_3$ (0.2 M) at room temperature (19–20 °C) (**C** & **D**). NMR sample preparation: The reaction mixture was centrifuged to allow precipitation/removal of most of the formed –rather insoluble in $CDCl_3$ **2a**– and the supernatant was subjected to NMR analysis.

## C) EtOCO-2ABA-OH + $SOCl_2$ study

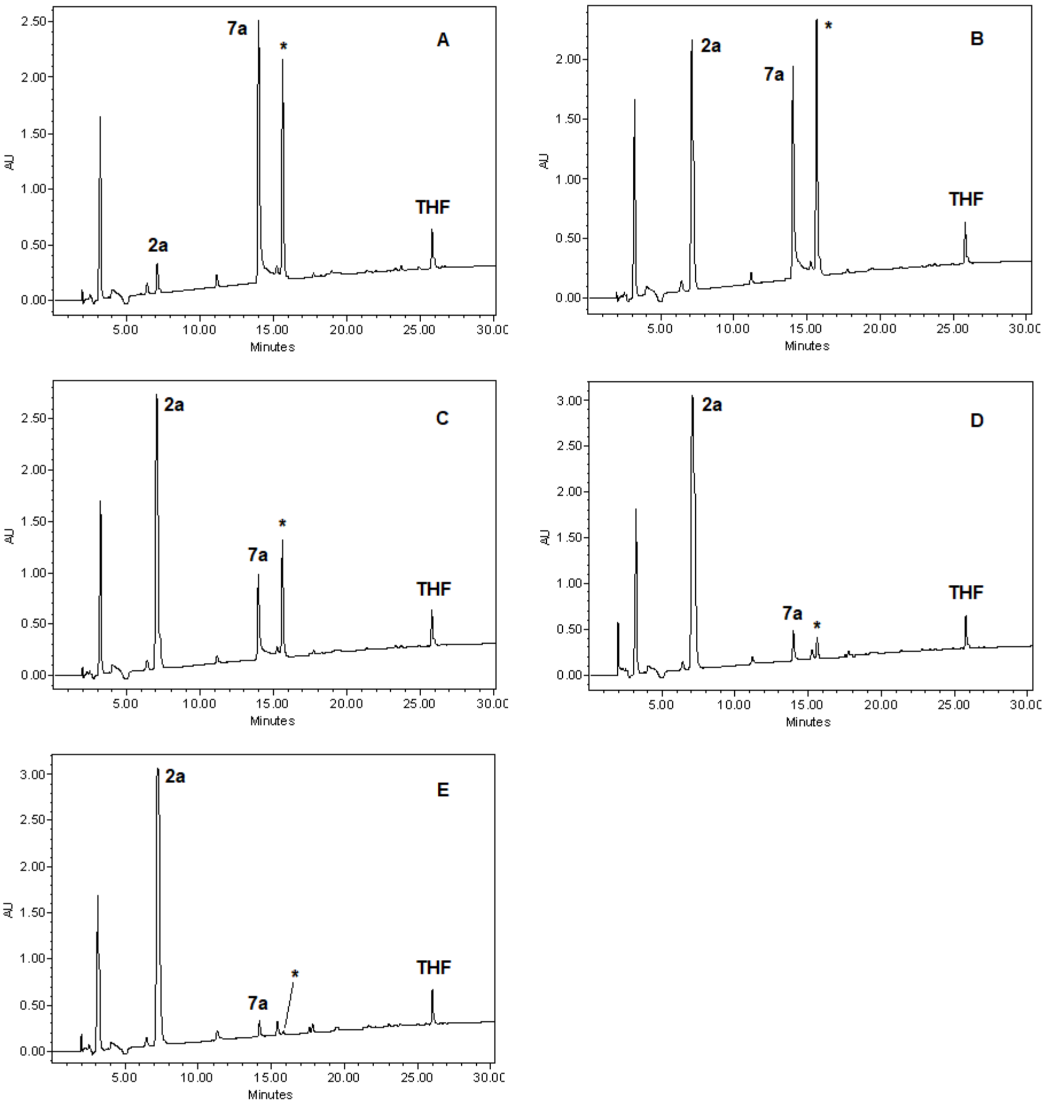


**Figure S7.** Representative hplc analysis for the conversion of **7a** to **2a** in presence of $SOCl_2$. Reaction conditions: 10 molar excess of $SOCl_2$ in respect to **7a** in THF (containing BHT as stabilizer) (0.2 M) at 16–17$^o$C. Hplc profile during the reaction mixture at 30 min (**A**), 1 h (**B**), 3 h (**C**), 5 h (**D**), 7 h (**E**). Hplc column/conditions: Puroshpere RP-8 (5μm); 250mm–4mm; 1.0 ml/min; 20% to 100% AcCN in 30 min; Abs: 214 nm.

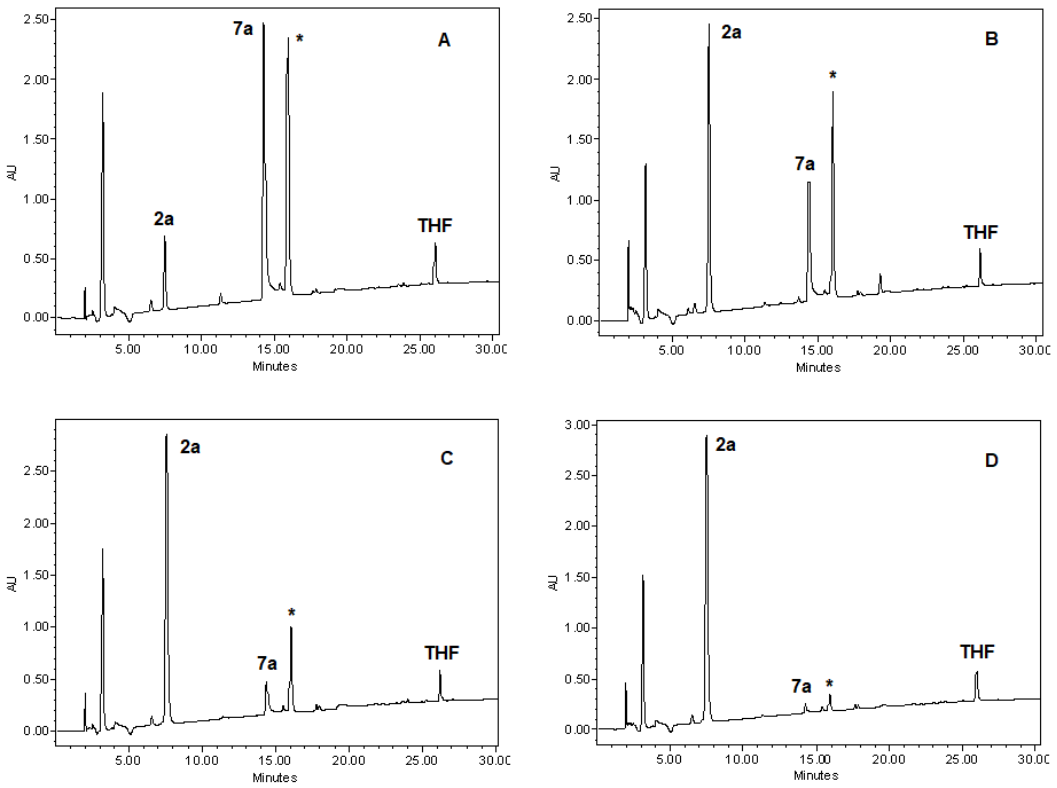


**Figure S8.** Representative hplc analysis for the conversion of **7a** to **2a** in presence of $SOCl_2$. Reaction conditions: 10 molar excess of $SOCl_2$ in respect to **7a** in THF (containing BHT as stabilizer) (0.2 M) at room temperature (22–24$^o$C). Hplc profile during the reaction mixture at 30 min (**A**), 1 h (**B**), 3 h (**C**), 4 h (**D**). Hplc column/conditions: Puroshpere RP-8 (5µm); 250mm–4mm; 1.0 ml/min; 20% to 100% AcCN in 30 min; Abs: 214 nm.

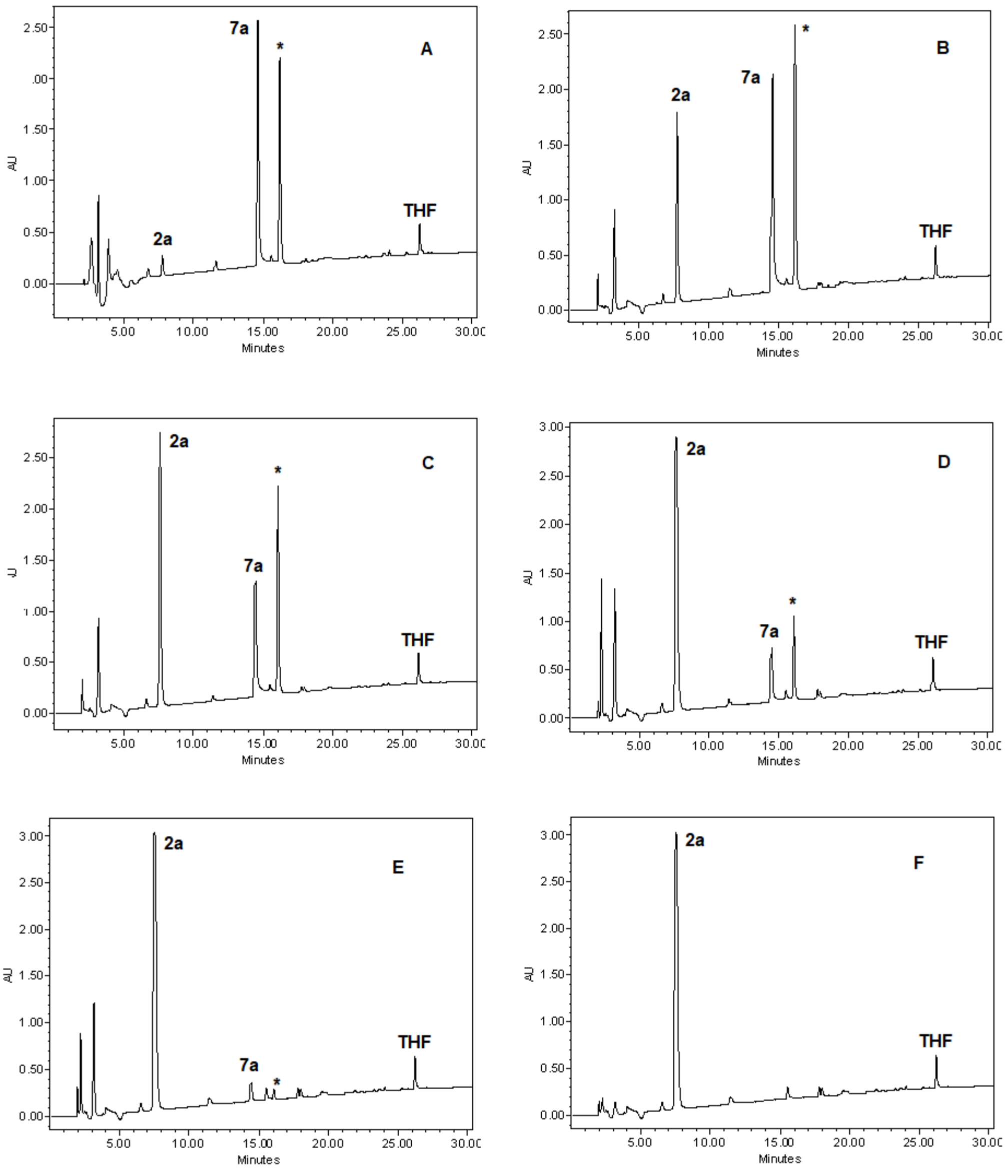


**Figure S9.** Representative hplc analysis for the conversion of **7a** to **2a** in presence of $SOCl_2$. Reaction conditions: 5 molar excess of $SOCl_2$ in respect to **7a** in THF (containing BHT as stabilizer) (0.2 M) at room temperature (22–24$^{o}$C). Hplc profile during the reaction mixture at 30 min (**A**), 1 h (**B**), 3 h (**C**), 5 h (**D**), 7 h (**E**), o.n. (**F**). Hplc column/conditions: Puroshpere RP-8 (5μm); 250mm–4mm; 1.0 ml/min; 20% to 100% AcCN in 30 min; Abs: 214 nm.

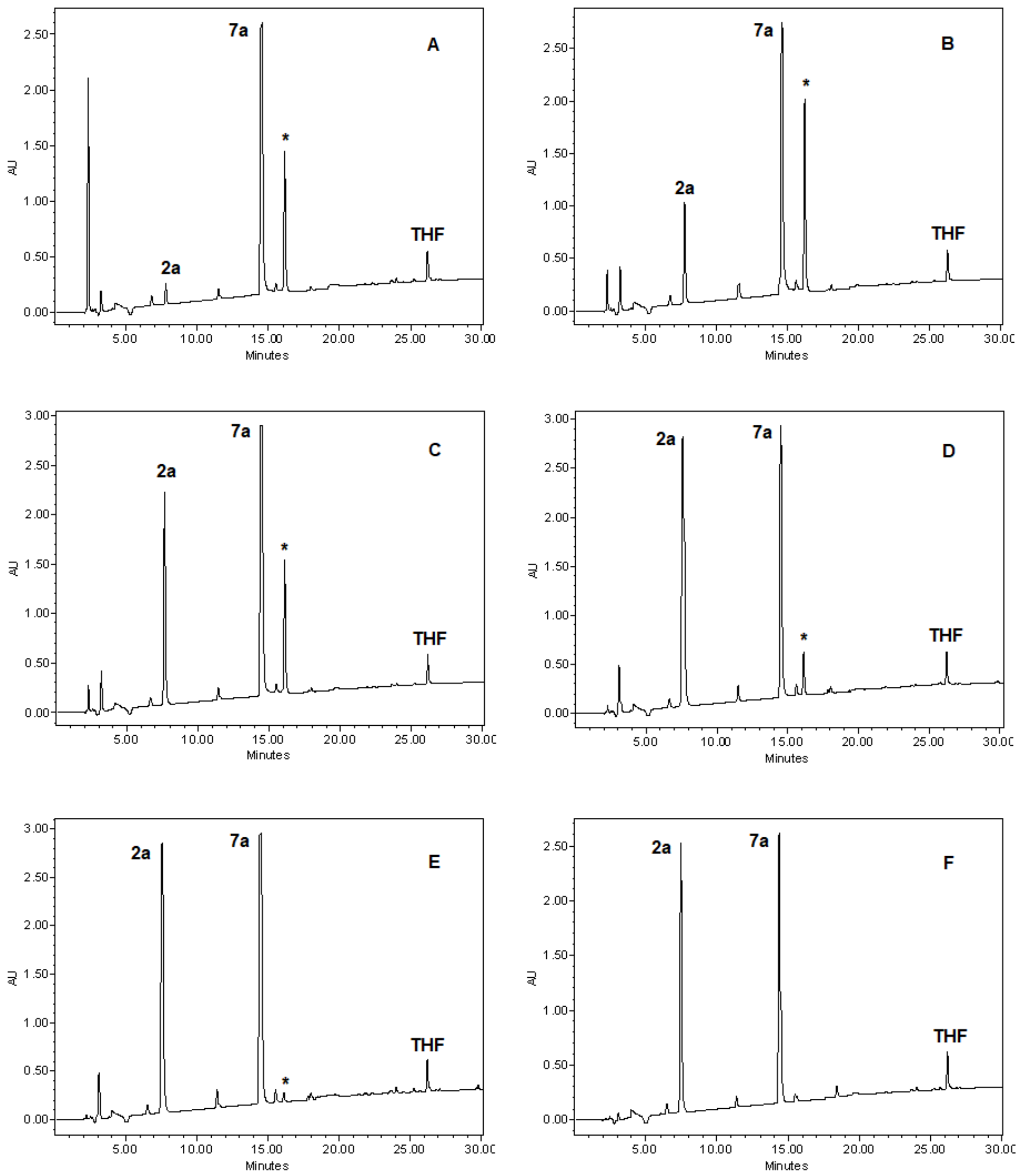


**Figure S10.** Representative hplc analysis for the conversion of **7a** to **2a** in presence of $SOCl_2$. Reaction conditions: 2 molar excess of $SOCl_2$ in respect to **7a** in THF (containing BHT as stabilizer) (0.2 M) at room temperature (22–24$^o$C). Hplc profile during the reaction mixture at 30 min (**A**), 1 h (**B**), 3 h (**C**), 5 h (**D**), 7 h (**E**) overnight (**F**). Hplc column/conditions: Puroshpere RP-8 (5µm); 250mm–4mm; 1.0 ml/min; 20% to 100% AcCN in 30 min; Abs: 214 nm.

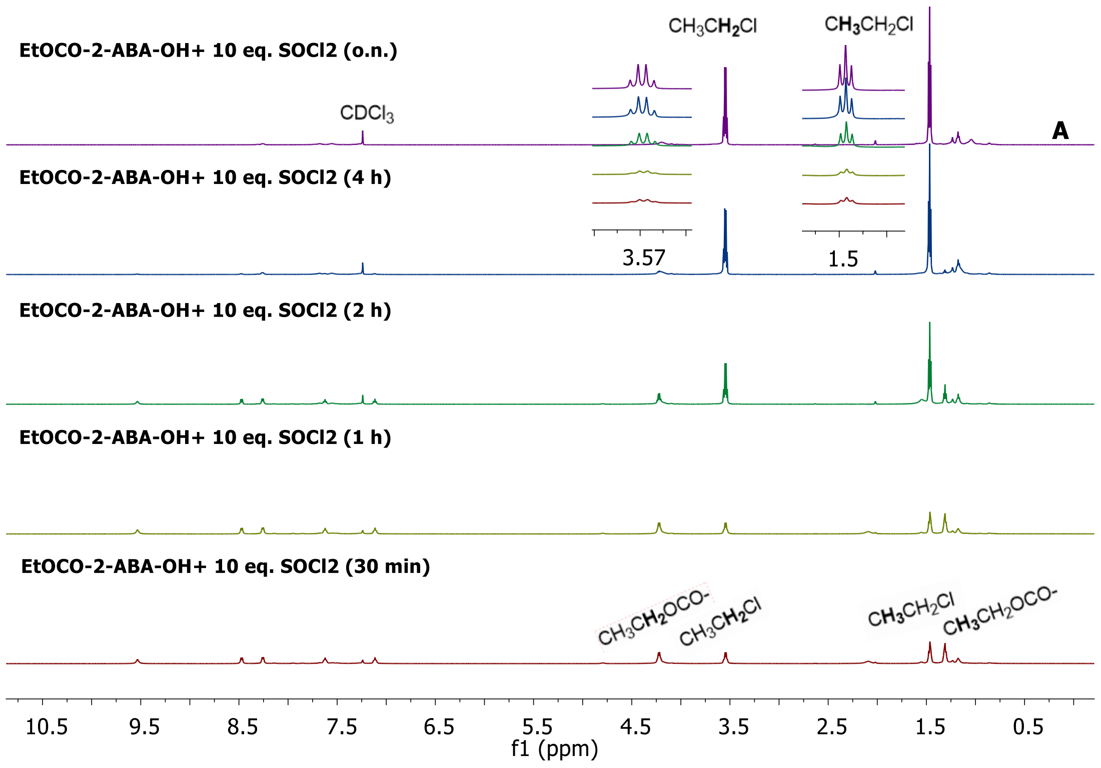
EtOCO-2-ABA-OH+ 10 eq. SOCl2 (o.n.)
EtOCO-2-ABA-OH+ 10 eq. SOCl2 (4 h)
EtOCO-2-ABA-OH+ 10 eq. SOCl2 (2 h)
EtOCO-2-ABA-OH+ 10 eq. SOCl2 (1 h)
EtOCO-2-ABA-OH+ 10 eq. SOCl2 (30 min)
$CDCl_3$
$CH_3\mathbf{CH_2}Cl$
$\mathbf{CH_3}CH_2Cl$
$CH_3\mathbf{CH_2}OCO-$
$\mathbf{CH_3}CH_2OCO-$
A
3.57
1.5
10.5
9.5
8.5
7.5
6.5
5.5
4.5
3.5
2.5
1.5
0.5
f1 (ppm)

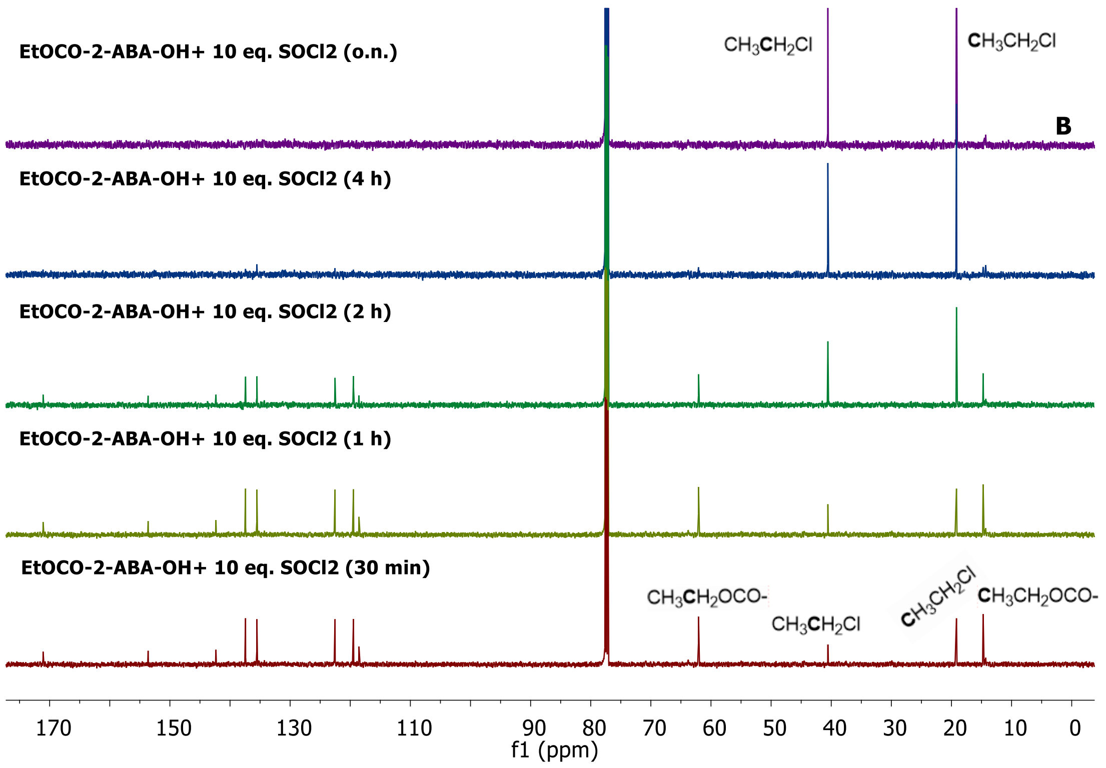
EtOCO-2-ABA-OH+ 10 eq. SOCl2 (o.n.)
EtOCO-2-ABA-OH+ 10 eq. SOCl2 (4 h)
EtOCO-2-ABA-OH+ 10 eq. SOCl2 (2 h)
EtOCO-2-ABA-OH+ 10 eq. SOCl2 (1 h)
EtOCO-2-ABA-OH+ 10 eq. SOCl2 (30 min)
$CH_3\mathbf{CH_2}Cl$
$\mathbf{CH_3}CH_2Cl$
$CH_3\mathbf{CH_2}OCO-$
$\mathbf{CH_3}CH_2OCO-$
B
170
150
130
110
90
80
70
60
50
40
30
20
10
0
f1 (ppm)

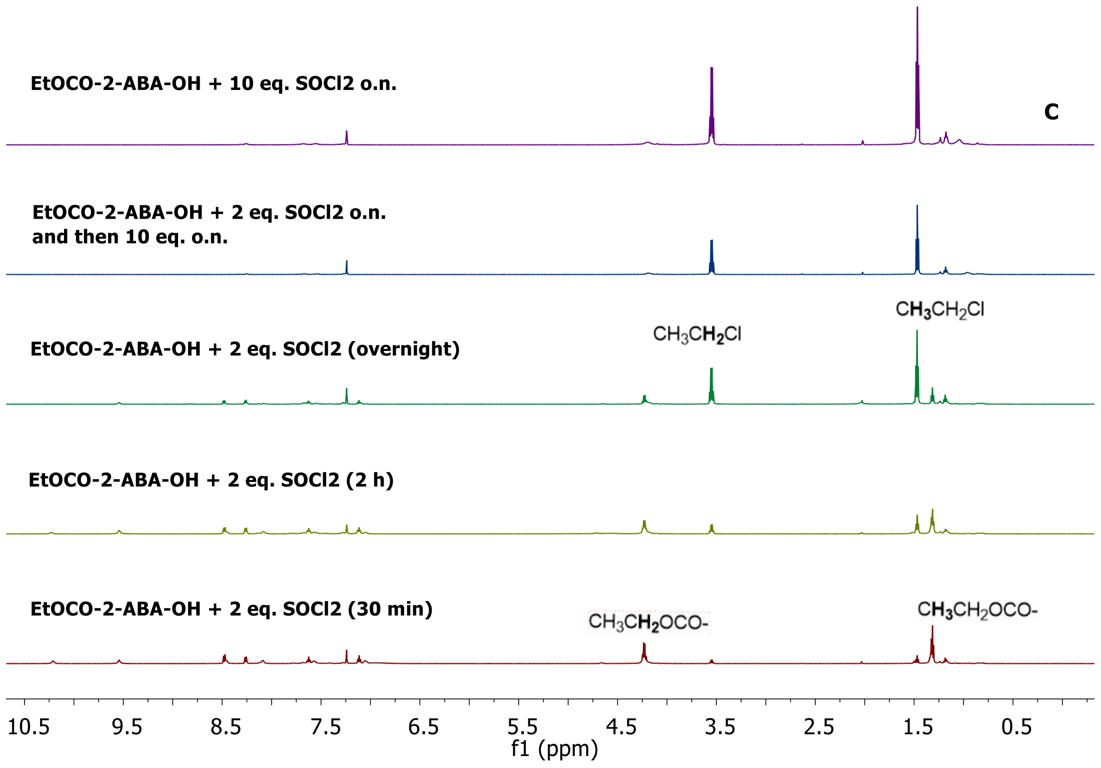


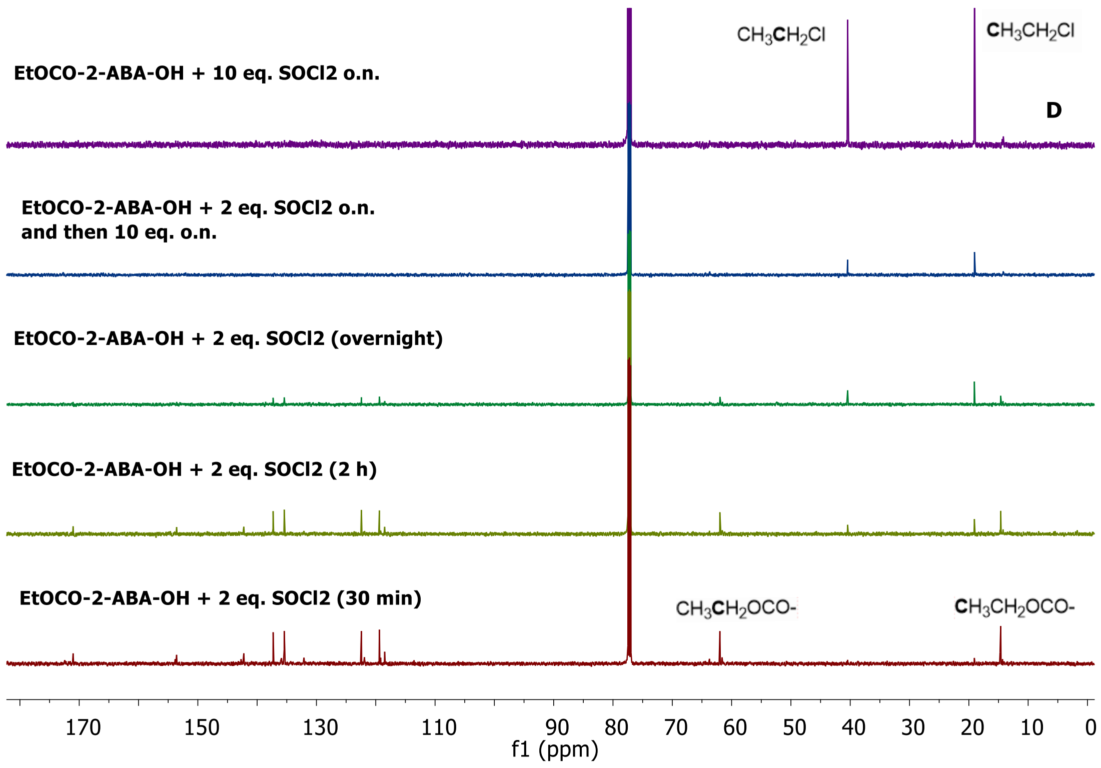


**Figure S11.** $^{1}H$-NMR and $^{13}C$-NMR of the reaction mixture of **7a** with $SOCl_2$, showing the formation of EtCl (**2a** was precipitated during its formation as white solid). Reaction conditions: 10 molar excess of $SOCl_2$ in respect to **7a** in $CDCl_3$ (0.2 M) at room temperature (19–20°C) (**A** & **B**); 2 molar excess of $SOCl_2$ in respect to **7a** in $CDCl_3$ (0.2 M) at room temperature (19–20 °C) (**C** & **D**). NMR sample preparation: $CDCl_3$ was added in **7a** and $SOCl_2$ was added. The initially non-dissolved **7a** was rapidly dissolved and the mixture was subjected to NMR analysis.

## D) NMR of Fmoc-, Cbz-, EtOCO-2-aminobenzoic acids 3a, 3b, 5a, 5b, 7a

**Fmoc-2-ABA-OH**

$^{1}$H NMR (600 MHz, DMSO-d) δ 10.79 (s, 1H), 8.15 (d, *J* = 5.3 Hz, 1H), 7.97 (d, *J* = 7.6 Hz, 1H), 7.91 (d, *J* = 7.4 Hz, 2H), 7.69 (d, *J* = 7.3 Hz, 2H), 7.57 (t, *J* = 7.4 Hz, 1H), 7.43 (t, *J* = 7.3 Hz, 2H), 7.34 (t, *J* = 7.2 Hz, 2H), 7.11 (t, *J* = 7.4 Hz, 1H), 4.49 (d, *J* = 6.6 Hz, 2H), 4.36 (t, *J* = 6.4 Hz, 1H).

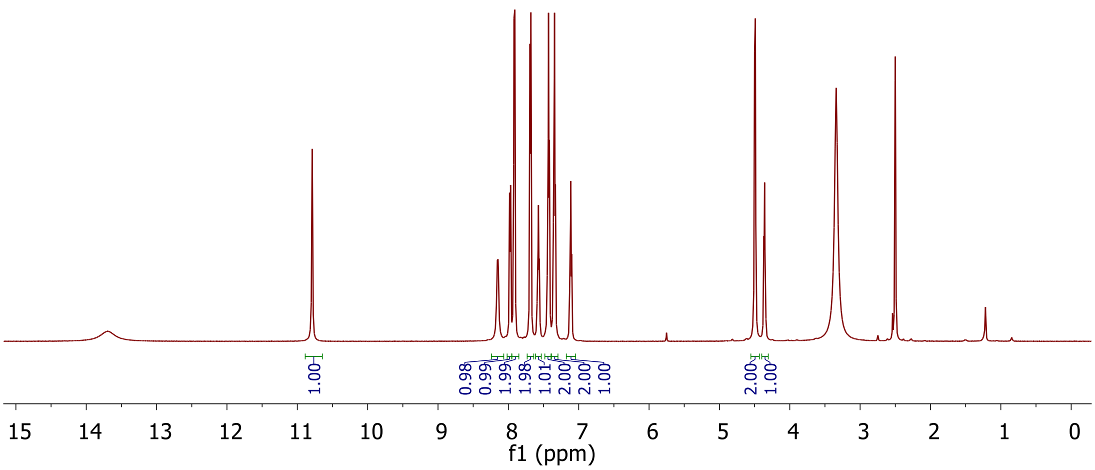


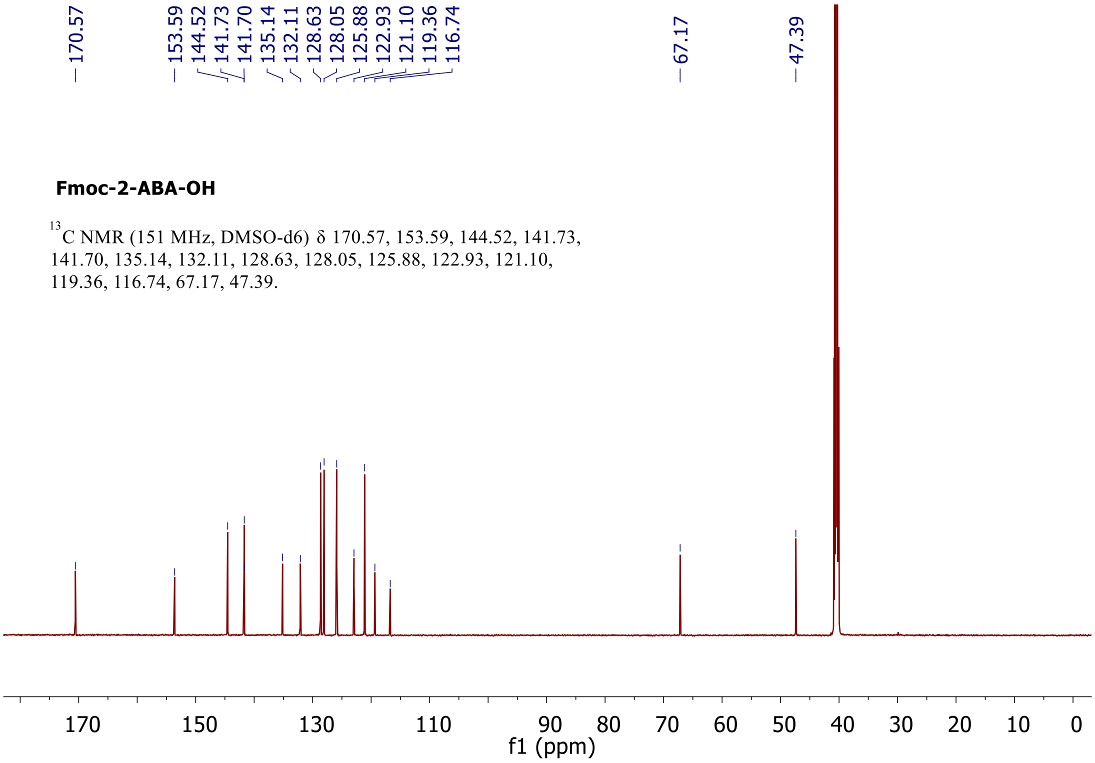


**Fmoc-2-ABA-OH**

$^{13}$C NMR (151 MHz, DMSO-d6) δ 170.57, 153.59, 144.52, 141.73, 141.70, 135.14, 132.11, 128.63, 128.05, 125.88, 122.93, 121.10, 119.36, 116.74, 67.17, 47.39.

**Figure S12: 3a** $^{1}$H-NMR *δ* (600 MHz, DMSO-d6) *δ* 13.70 (s), 10.81 (s), 8.15 (d, *J* = 5.6 Hz), 7.98 (d, *J* = 7.7 Hz), 7.92 (d, *J* = 7.4 Hz), 7.69 (d, *J* = 7.3 Hz), 7.57 (t, *J* = 7.5 Hz), 7.43 (t, *J* = 7.5 Hz), 7.35 (t, *J* = 7.4 Hz), 7.11 (t, *J* = 7.5 Hz), 4.49 (d, *J* = 6.7 Hz), 4.36 (t, *J* = 6.6 Hz); $^{13}$C NMR *δ* (151 MHz, DMSO-d6) δ 170.57, 153.59, 144.52, 141.73, 141.70, 135.14, 132.11, 128.63, 128.05, 125.88, 122.93, 121.10, 119.36, 116.74, 67.17, 47.39.

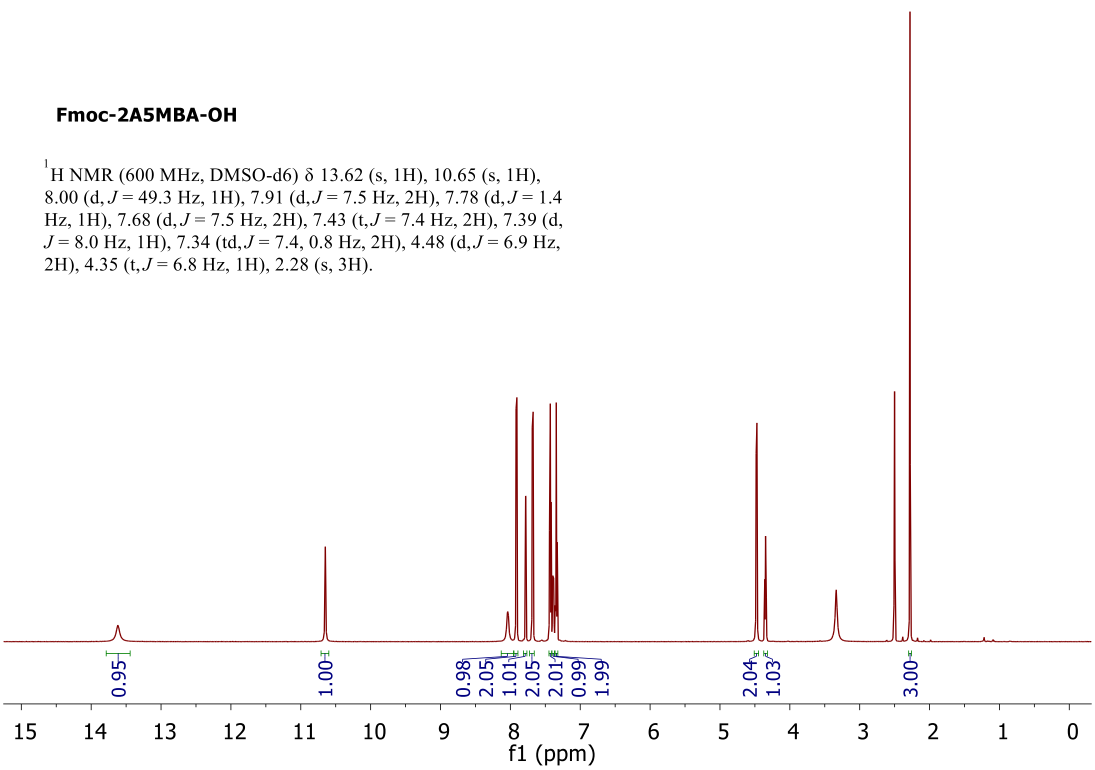


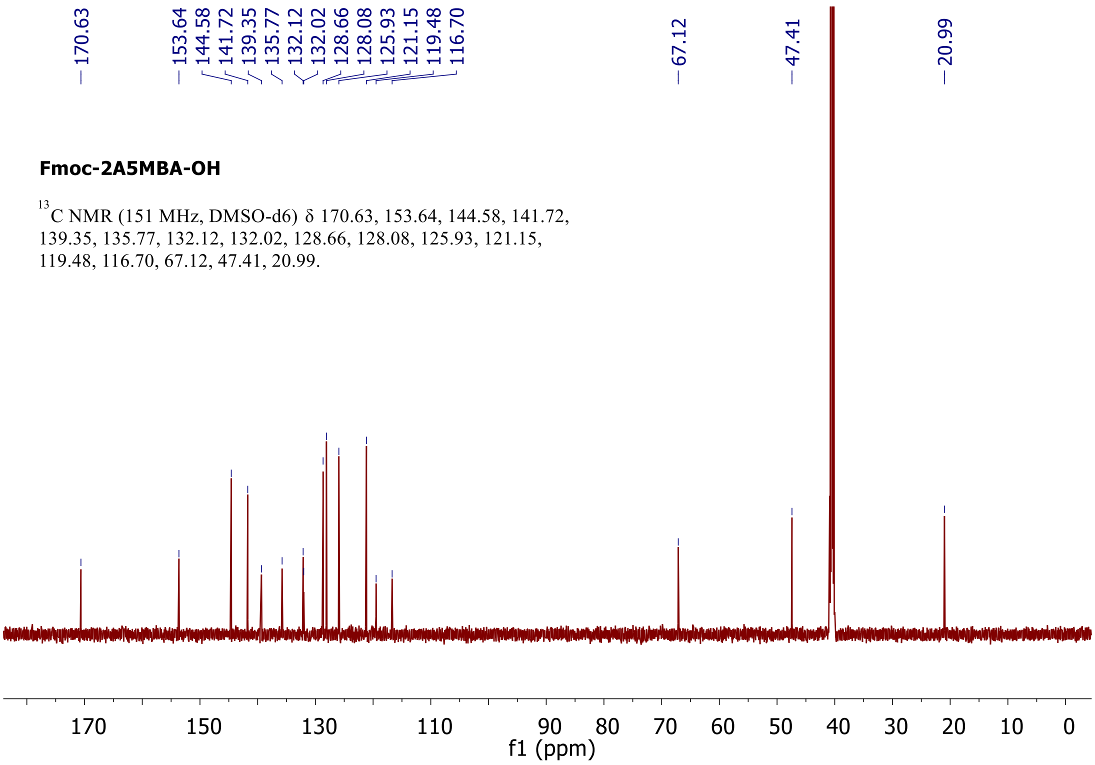


**Figure S13: 3b** $^{1}H$ NMR *δ* (600 MHz, DMSO-$d_6$) *δ* 13.62 (s, 1H), 10.65 (s, 1H), 8.00 (d, *J* = 49.3 Hz, 1H), 7.91 (d, *J* = 7.5 Hz, 2H), 7.78 (d, *J* = 1.4 Hz, 1H), 7.68 (d, *J* = 7.5 Hz, 2H), 7.43 (t, *J* = 7.4 Hz, 2H), 7.39 (d, *J* = 8.0 Hz, 1H), 7.34 (td, *J* = 7.4, 0.8 Hz, 2H), 4.48 (d, *J* = 6.9 Hz, 2H), 4.35 (t, *J* = 6.8 Hz, 1H), 2.28 (s, 3H) ; $^{13}C$ NMR *δ* (151 MHz, DMSO-$d_6$) *δ* 170.63, 153.64, 144.58, 141.72, 139.35, 135.77, 132.12, 132.02, 128.66, 128.08, 125.93, 121.15, 119.48, 116.70, 67.12, 47.41, 20.99.

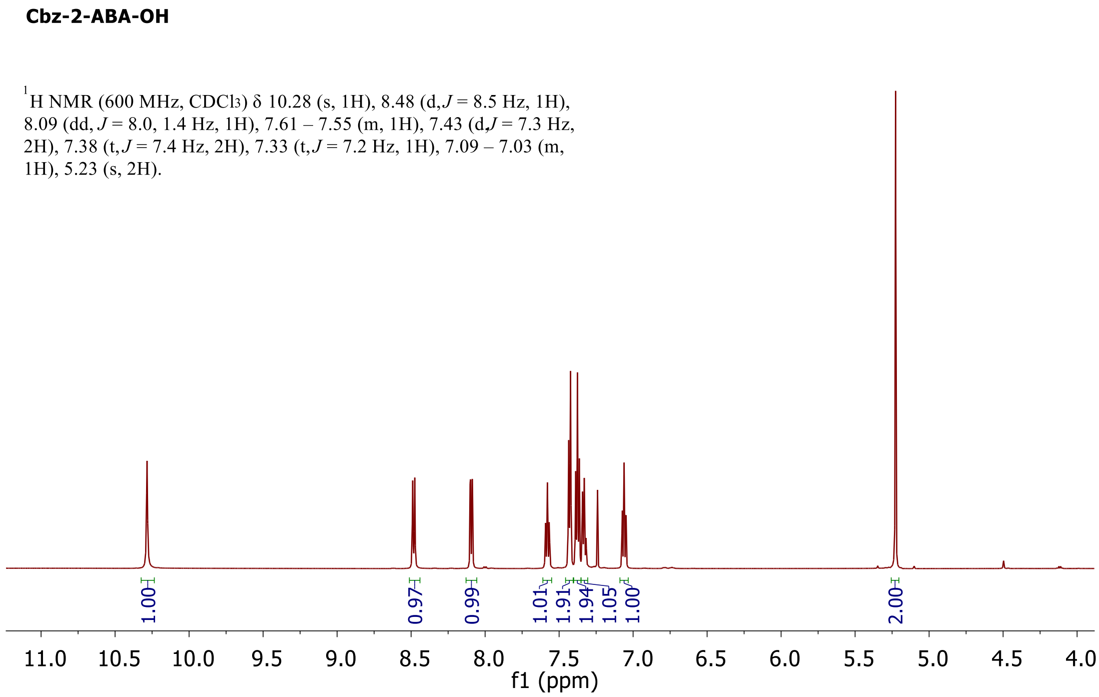


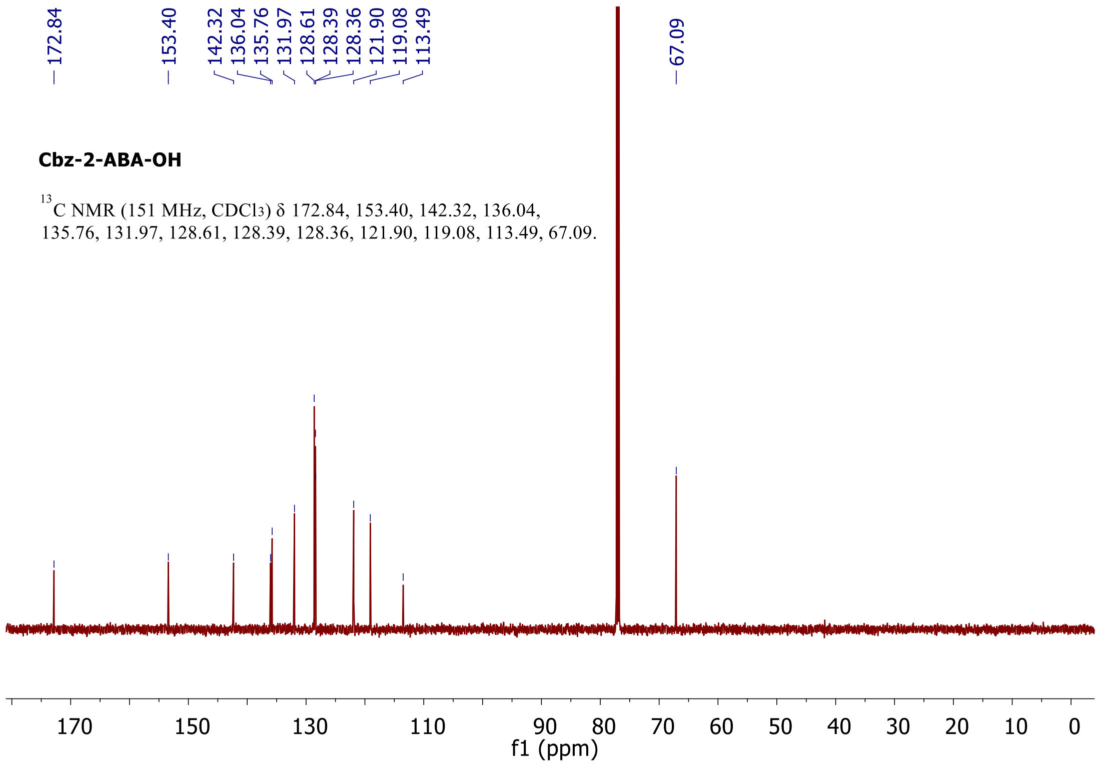


**Figure S14: 5a** $^{1}H$ NMR (600 MHz, $CDCl_3$) $\delta$ 10.28 (s, 1H), 8.48 (d, $J$ = 8.5 Hz, 1H), 8.09 (dd, $J$ = 8.0, 1.4 Hz, 1H), 7.61 – 7.55 (m, 1H), 7.43 (d, $J$ = 7.3 Hz, 2H), 7.38 (t, $J$ = 7.4 Hz, 2H), 7.33 (t, $J$ = 7.2 Hz, 1H), 7.09 – 7.03 (m, 1H), 5.23 (s, 2H); $^{13}C$ NMR $\delta$ (151 MHz, $CDCl_3$) δ 172.84, 153.40, 142.32, 136.04, 135.76, 131.97, 128.61, 128.39, 128.36, 121.90, 119.08, 113.49, 67.09.

**Cbz-2A5MBA-OH**

$^{1}$H NMR (600 MHz, MeOH-d4) δ 8.24 (d, *J* = 8.4 Hz, 1H), 7.86 (s, 1H), 7.49 – 7.20 (m, 6H), 5.19 (s, 2H), 2.31 (s, 3H).

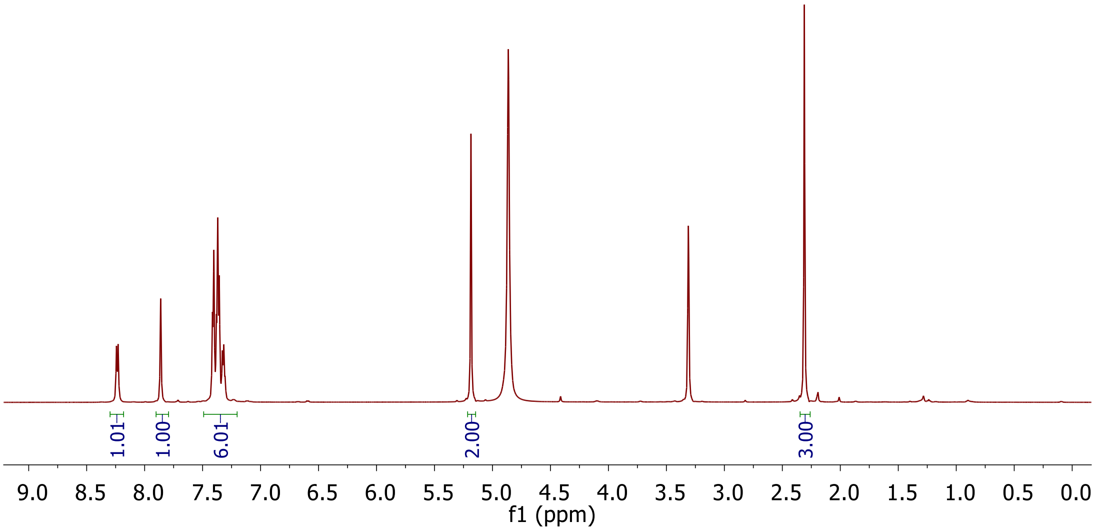


**Cbz-2A5MBA-OH**

$^{13}$C NMR (151 MHz, MeOH-d4) δ 172.22, 155.80, 141.31, 138.73, 136.85, 133.47, 133.42, 130.43, 130.08, 129.97, 120.54, 117.33, 68.65, 21.41.

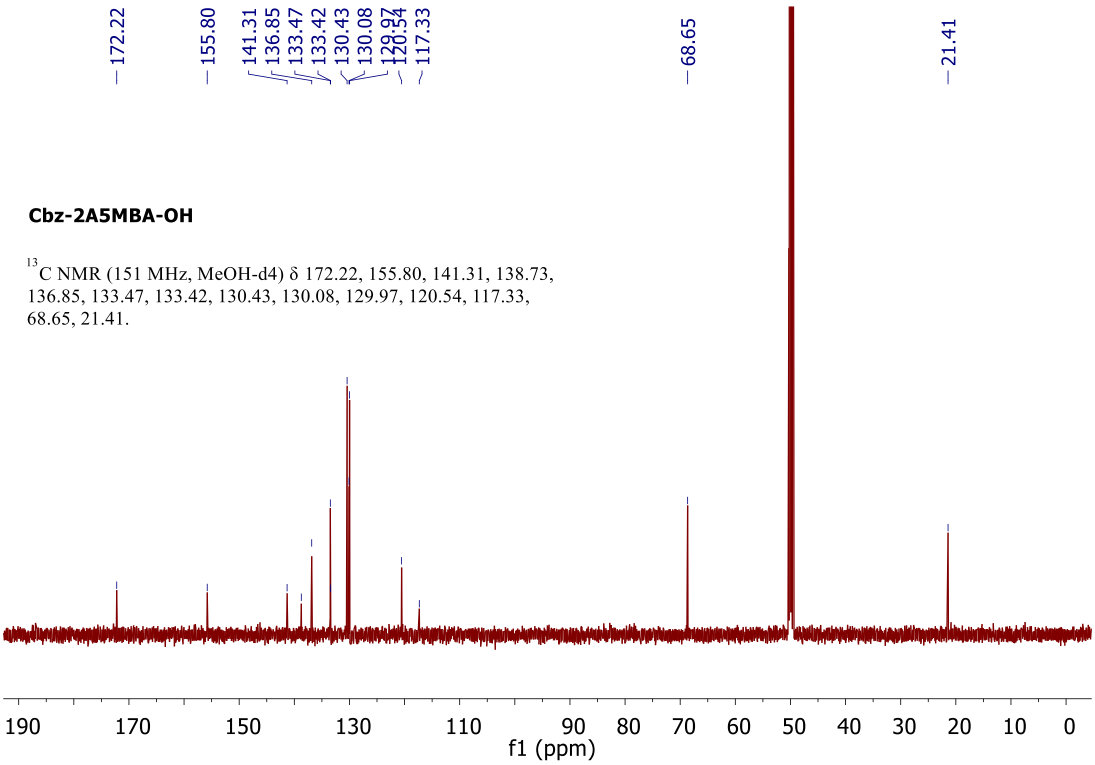


**Figure S15: 5b** $^1$H NMR (600 MHz, $CD_3OD$) *δ* 8.24 (d, *J* = 8.4 Hz, 1H), 7.86 (s, 1H), 7.49 – 7.20 (m, 6H), 5.19 (s, 2H), 2.31 (s, 3H) ; $^{13}$C NMR (151 MHz, $CD_3OD$) *δ* 172.22, 155.80, 141.31, 138.73, 136.85, 133.47, 133.42, 130.43, 130.08, 129.97, 120.54, 117.33, 68.65, 21.41.

**EtOCO-2-ABA-OH**

$^{1}$H NMR (600 MHz, DMSO-d6) δ 13.48 (s, 1H), 8.13 (d,*J* = 8.1 Hz, 1H), 7.96 (d, *J* = 7.3 Hz, 1H), 7.27 (t,*J* = 7.3 Hz, 1H), 6.89 (t,*J* = 7.3 Hz, 1H), 4.09 (dd, *J* = 14.0, 7.0 Hz, 2H), 1.22 (t,*J* = 7.0 Hz, 3H).

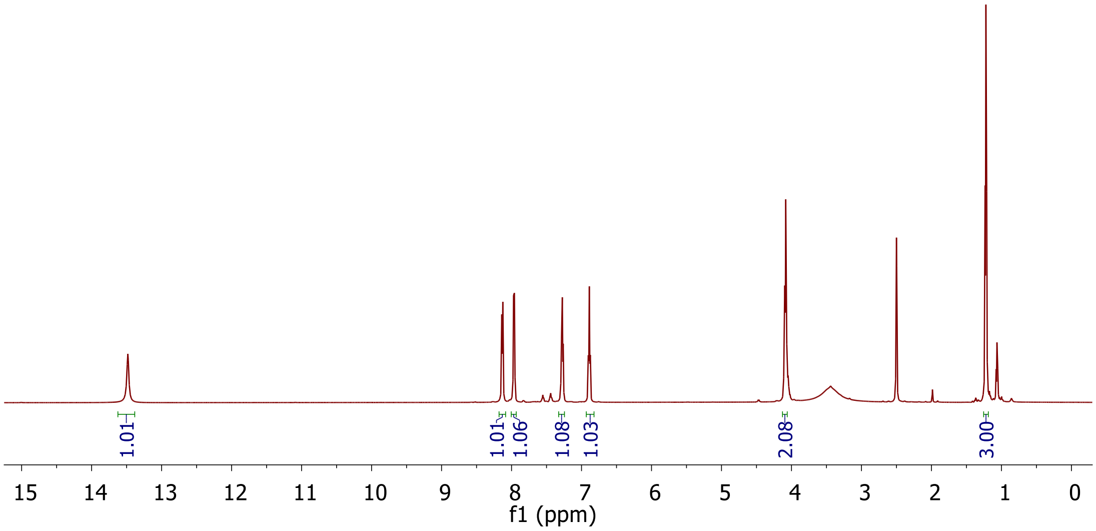


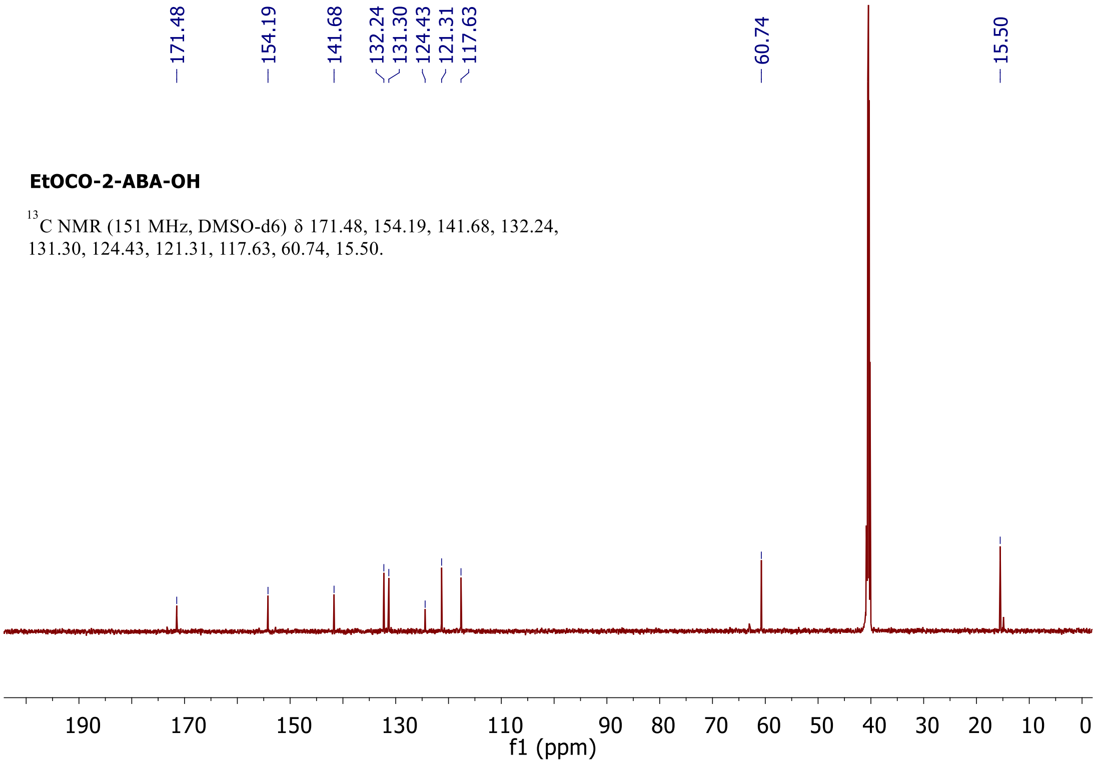


**Figure S16: 7a** $^{1}$H NMR (600 MHz, DMSO-$d_6$) $\delta$ 13.48 (s, 1H), 8.13 (d, *J* = 8.1 Hz, 1H), 7.96 (d, *J* = 7.3 Hz, 1H), 7.27 (t, *J* = 7.3 Hz, 1H), 6.89 (t, *J* = 7.3 Hz, 1H), 4.09 (dd, *J* = 14.0, 7.0 Hz, 2H), 1.22 (t, *J* = 7.0 Hz, 3H); $^{13}$C NMR (151 MHz, DMSO-$d_6$) $\delta$ 171.48, 154.19, 141.68, 132.24, 131.30, 124.43, 121.31, 117.63, 60.74, 15.50.

## E) NMR of final products 2a–2f

**1H-benzo[*d*][1,3]oxazine-2,4-dione**

$^{1}$H NMR (600 MHz, DMSO-d6) δ 11.72 (s, 1H), 7.92 (dd, *J* = 7.9, 1.0 Hz, 1H), 7.77 – 7.71 (m, 1H), 7.28 – 7.22 (m, 1H), 7.15 (d, *J* = 8.2 Hz, 1H).

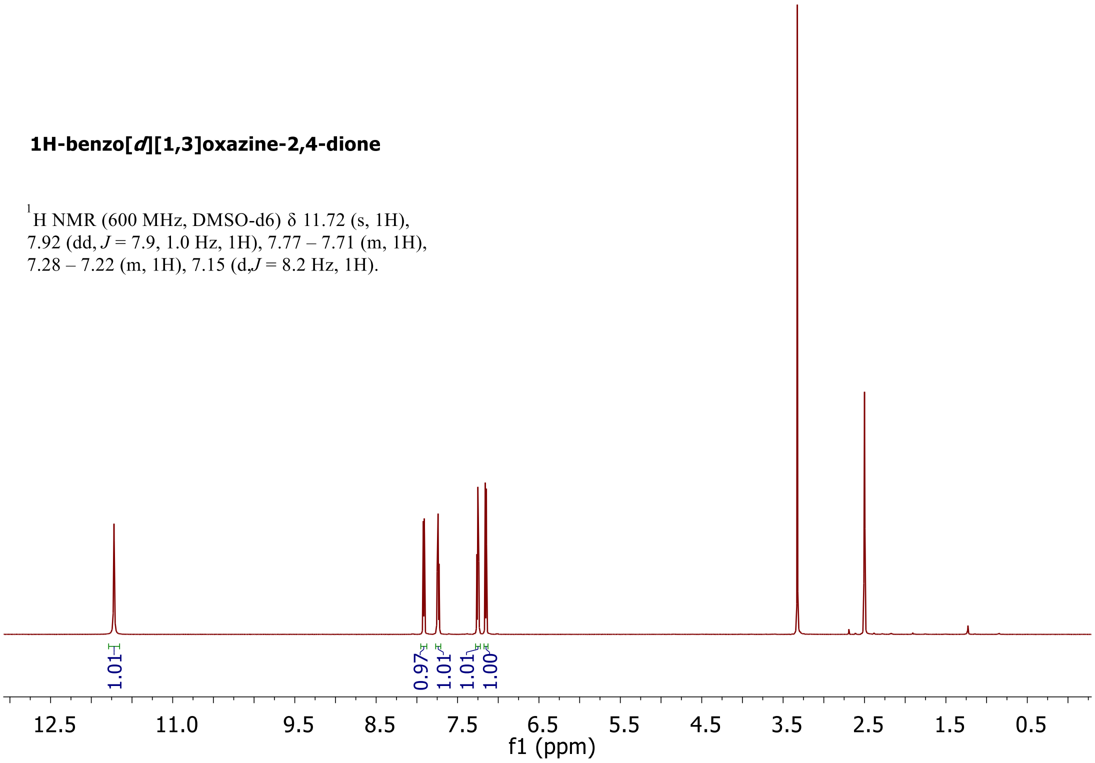


**1H-benzo[*d*][1,3]oxazine-2,4-dione**

$^{13}$C NMR (151 MHz, DMSO-d6) δ 160.81, 148.02, 142.33, 137.86, 129.86, 124.44, 116.26, 116.23, 111.20.

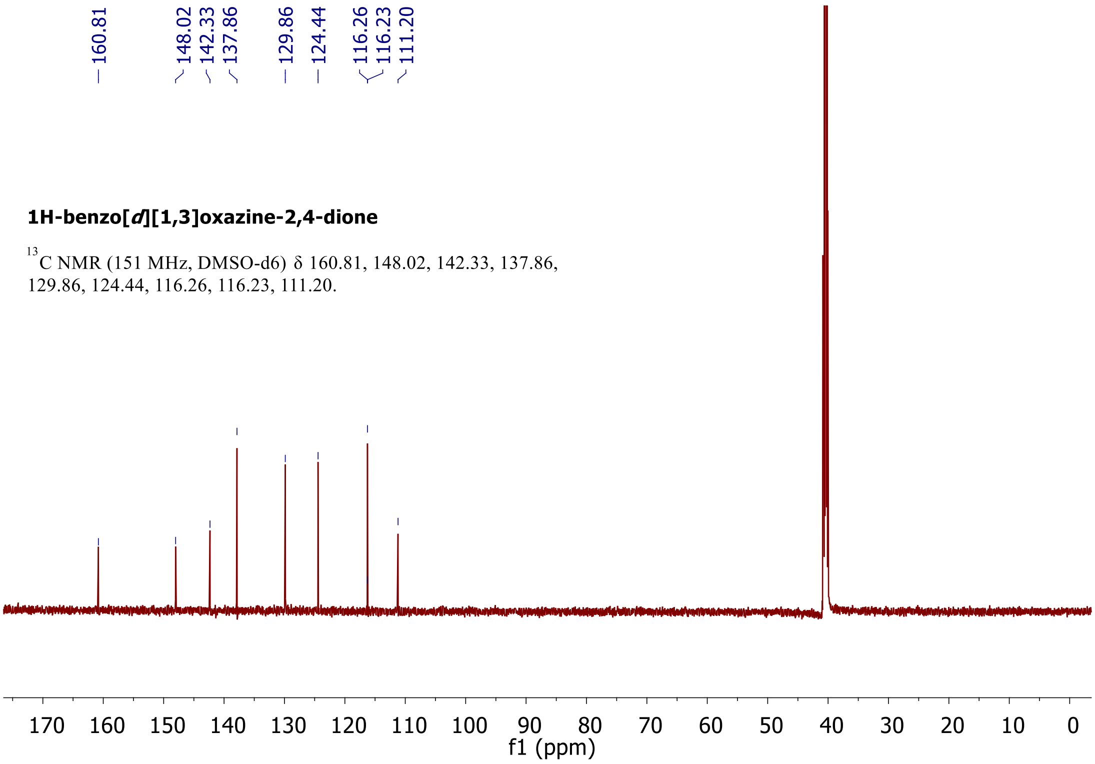


**Figure S17: 2a** $^{1}$H NMR (600 MHz, DMSO-d$_6$) $\delta$ 11.72 (s, 1H), 7.92 (dd, *J* = 7.9, 1.0 Hz, 1H), 7.77 – 7.71 (m, 1H), 7.28 – 7.22 (m, 1H), 7.15 (d, *J* = 8.2 Hz, 1H); $^{13}$C NMR (151 MHz, DMSO-d$_6$) $\delta$ 160.81, 148.02, 142.33, 137.86, 129.86, 124.44, 116.26, 116.23, 111.20.

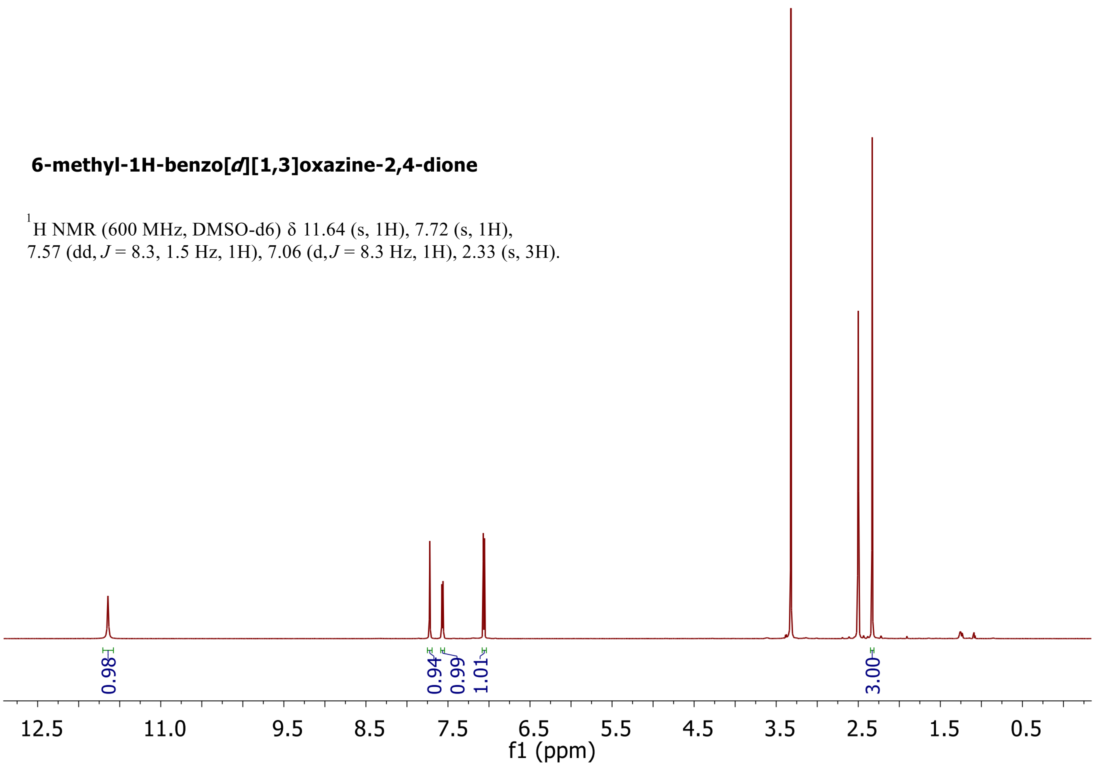


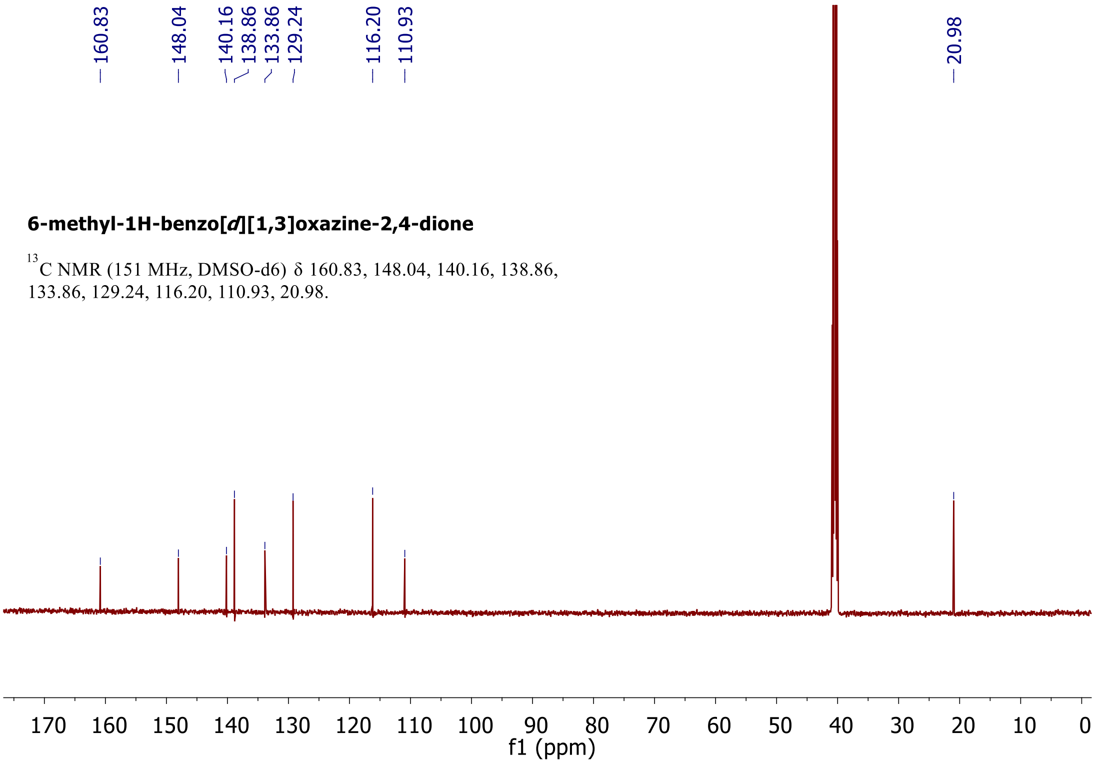


**Figure S18: 2b** $^{1}H$ NMR (600 MHz, DMSO-$d_6$) $\delta$ 11.64 (s, 1H), 7.72 (s, 1H), 7.57 (dd, $J$ = 8.3, 1.5 Hz, 1H), 7.06 (d, $J$ = 8.3 Hz, 1H), 2.33 (s, 3H); $^{13}C$ NMR (151 MHz, DMSO-$d_6$) $\delta$ 160.83, 148.04, 140.16, 138.86, 133.86, 129.24, 116.20, 110.93, 20.98.

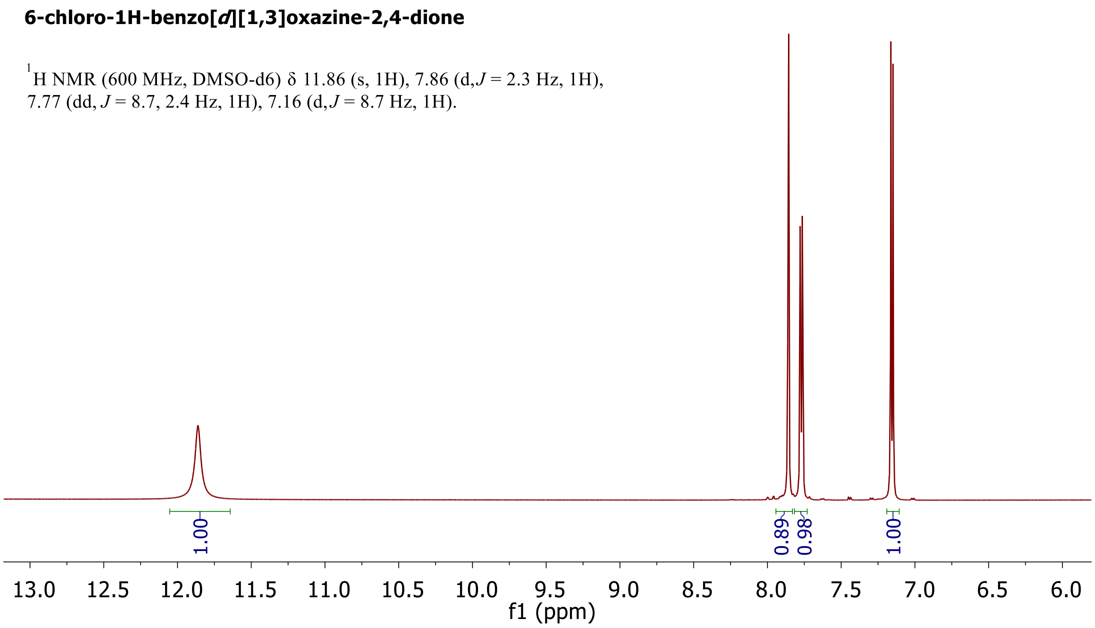


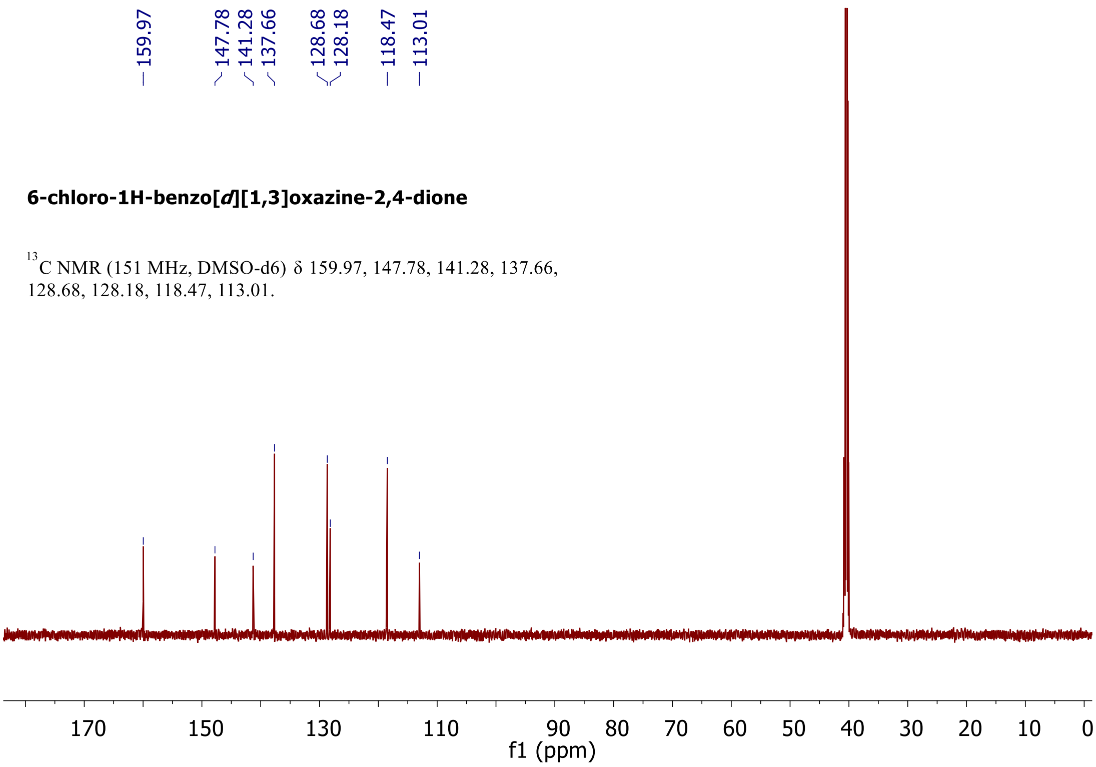


**Figure S19: 2c** $^{1}H$ NMR (600 MHz, DMSO-$d_6$) $\delta$ 11.86 (s, 1H), 7.86 (d, $J$ = 2.3 Hz, 1H), 7.77 (dd, $J$ = 8.7, 2.4 Hz, 1H), 7.16 (d, $J$ = 8.7 Hz, 1H) ; $^{13}C$ NMR (151 MHz, DMSO-$d_6$) $\delta$ 159.97, 147.78, 141.28, 137.66, 128.68, 128.18, 118.47, 113.01.

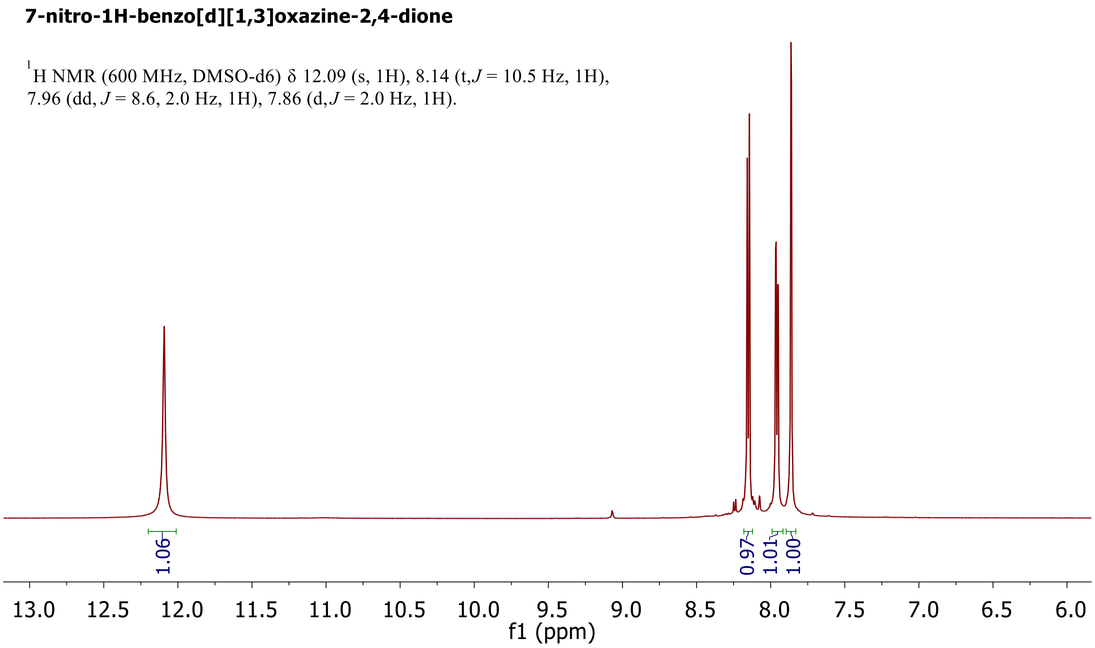


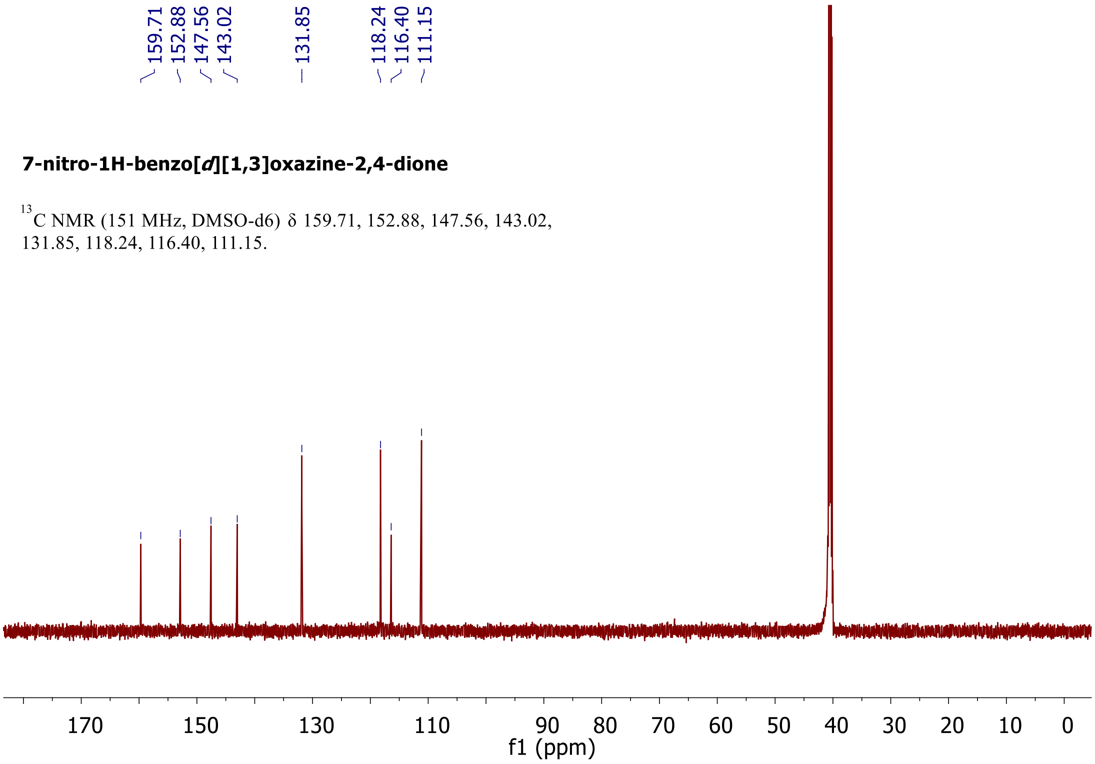


**Figure S20: 2d** $^{1}$H NMR (600 MHz, DMSO-$d_6$) $\delta$ 12.09 (s, 1H), 8.14 (t, $J$ = 10.5 Hz, 1H), 7.96 (dd, $J$ = 8.6, 2.0 Hz, 1H), 7.86 (d, $J$ = 2.0 Hz, 1H) ; $^{13}$C NMR (151 MHz, DMSO-$d_6$) $\delta$ 159.71, 152.88, 147.56, 143.02, 131.85, 118.24, 116.40, 111.15.

**6-nitro-1H-benzo[*d*][1,3]oxazine-2,4-dione**

$^{1}$H NMR (600 MHz, DMSO-d6) δ 12.36 (s, 1H), 8.56 (dd, *J* = 10.7, 4.3 Hz, 1H), 8.52 (dd, *J* = 9.0, 2.6 Hz, 1H), 7.33 (t, *J* = 11.8 Hz, 1H).

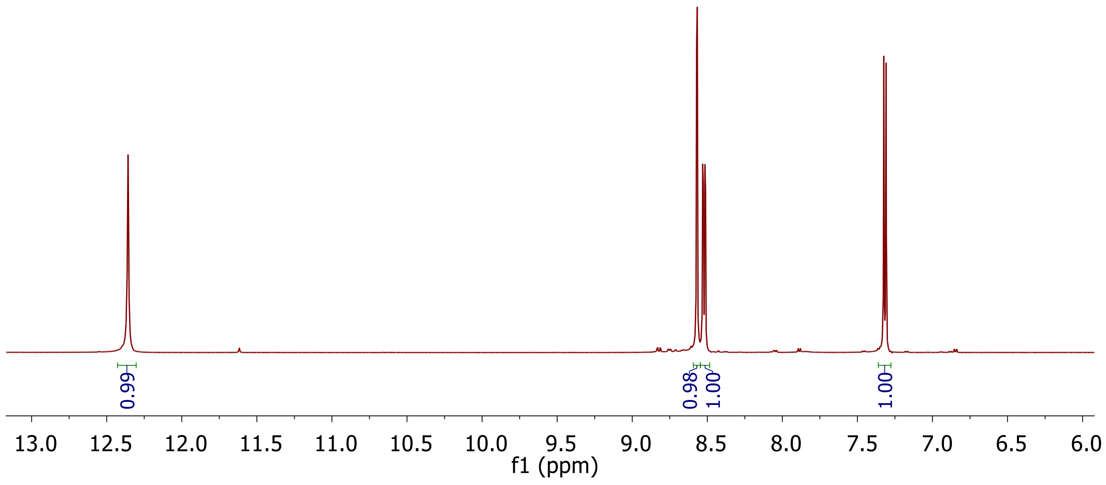


**6-nitro-1H-benzo[*d*][1,3]oxazine-2,4-dione**

$^{13}$C NMR (151 MHz, DMSO-d6) δ 159.56, 147.43, 146.99, 143.36, 132.17, 125.47, 117.60, 112.05.

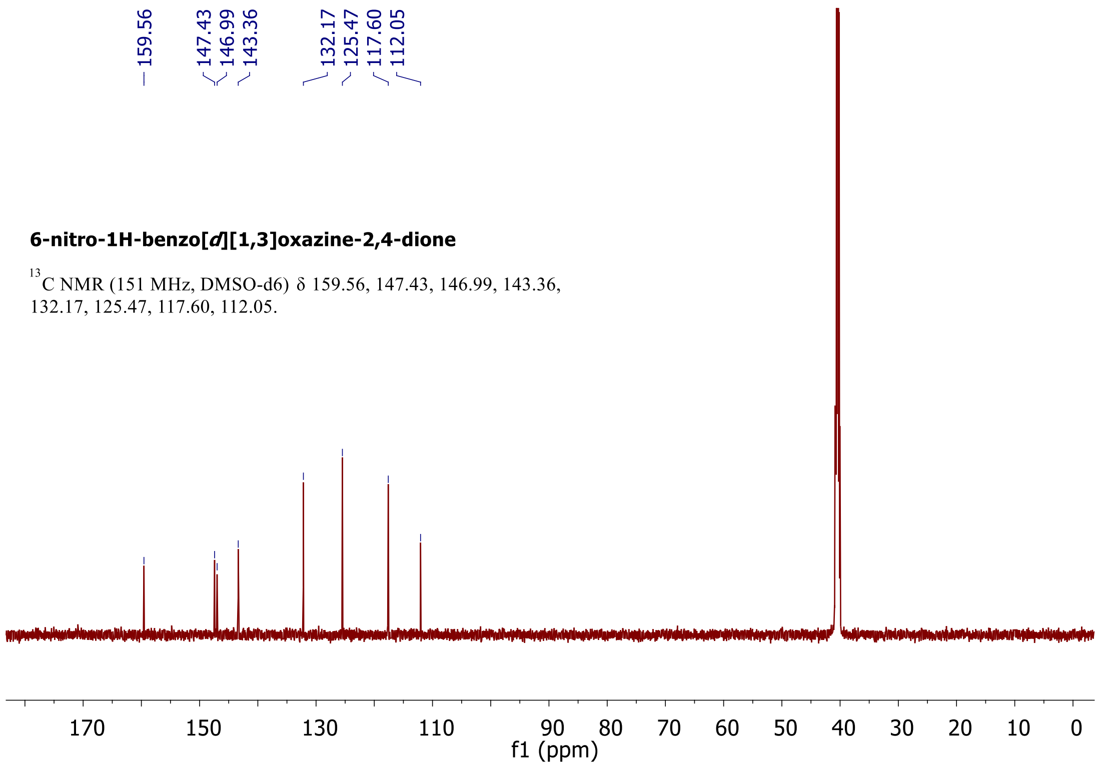


**Figure S21: 2e** $^{1}$H NMR (600 MHz, DMSO-d6) $\delta$ 12.36 (s, 1H), 8.56 (dd, *J* = 10.7, 4.3 Hz, 1H), 8.52 (dd, *J* = 9.0, 2.6 Hz, 1H), 7.33 (t, *J* = 11.8 Hz, 1H) ; $^{13}$C NMR (151 MHz, DMSO-$d_6$) $\delta$ 159.56, 147.43, 146.99, 143.36, 132.17, 125.47, 117.60, 112.05.

**1H-naphtho[2,3-d][1,3]oxazine-2,4-dione**

$^1$H NMR (600 MHz, DMSO-d6) δ 11.77 (s, 1H), 8.69 (d, *J* = 16.0 Hz, 1H), 8.11 (d, *J* = 8.2 Hz, 1H), 7.92 (d, *J* = 8.3 Hz, 1H), 7.65 (dd, *J* = 16.6, 9.2 Hz, 1H), 7.52 – 7.44 (m, 2H).

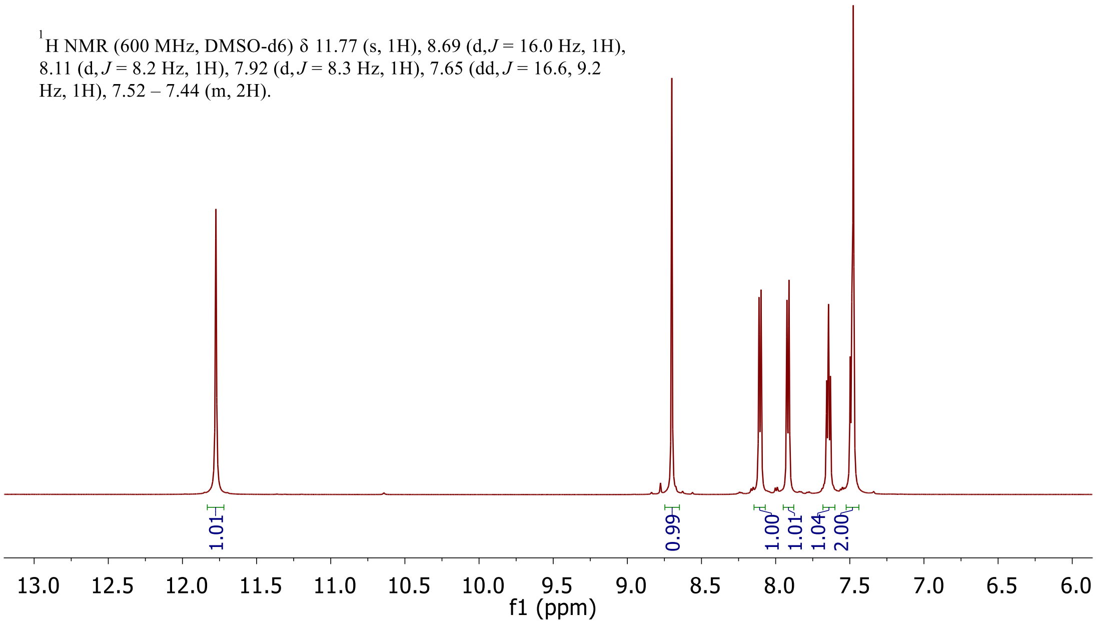


**1H-naphtho[2,3-d][1,3]oxazine-2,4-dione**

$^{13}$C NMR (151 MHz, DMSO-d6) δ 160.90, 147.86, 137.90, 136.91, 132.67, 131.11, 130.62, 129.80, 127.82, 126.41, 112.00, 111.33.

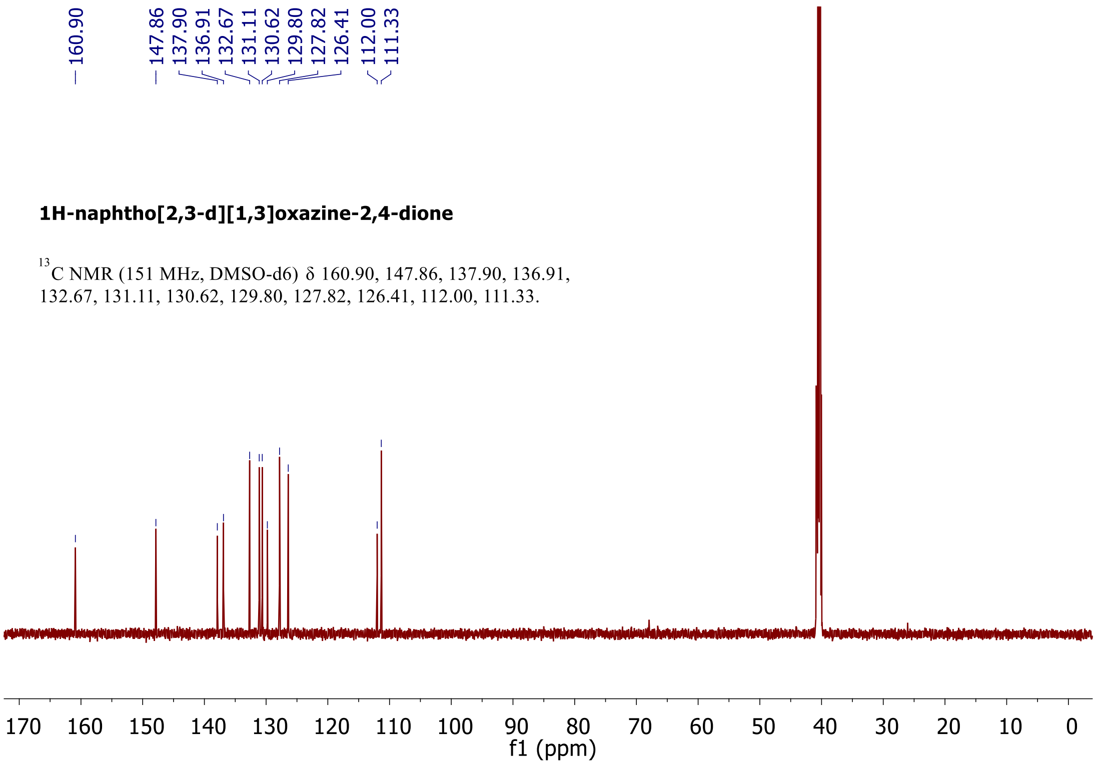


**Figure S22: 2f** $^1$H NMR (600 MHz, DMSO-$d_6$) $\delta$ 11.77 (s, 1H), 8.69 (d, *J* = 16.0 Hz, 1H), 8.11 (d, *J* = 8.2 Hz, 1H), 7.92 (d, *J* = 8.3 Hz, 1H), 7.65 (dd, *J* = 16.6, 9.2 Hz, 1H), 7.52 – 7.44 (m, 2H) ; $^{13}$C NMR (151 MHz, DMSO-$d_6$) $\delta$ 160.90, 147.86, 137.90, 136.91, 132.67, 131.11, 130.62, 129.80, 127.82, 126.41, 112.00, 111.33.

## F) HPLC analysis of final products 2a-f

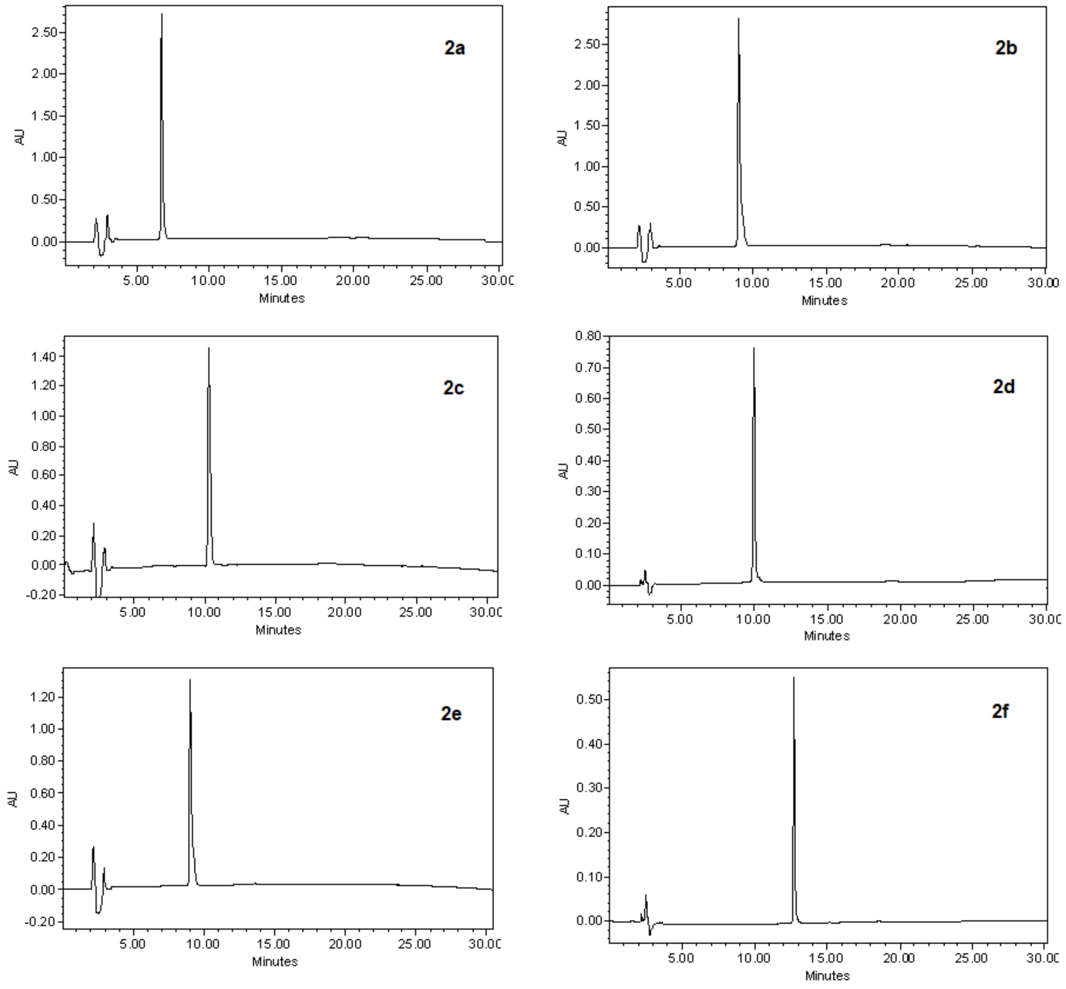


**Figure S23:** Hplc analysis of **2a-f**. Hplc column/conditions: Puroshpere RP-8 (5µm); 250mm–4mm; 1.0 ml/min; 20% to 100% AcCN in 30 min; Abs: 214 nm.

## G) NMR and HPLC analysis of 2-ethoxy-4*H*-benzo[*d*][1,3]oxazin-4-one

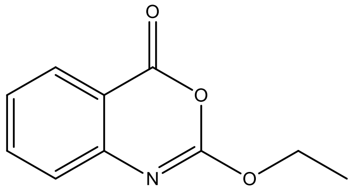


**2-ethoxy-4*H*-benzo[*d*][1,3]oxazin-4-one**

**A**

$^{1}$H NMR (600 MHz, CDCl3) δ 8.09 (dd, *J* = 7.9, 1.2 Hz, 1H), 7.74 – 7.64 (m, 1H), 7.39 (d, *J* = 8.1 Hz, 1H), 7.31 (dd, *J* = 11.2, 3.9 Hz, 1H), 4.50 (q, *J* = 7.1 Hz, 2H), 1.43 (t, *J* = 7.1 Hz, 3H).

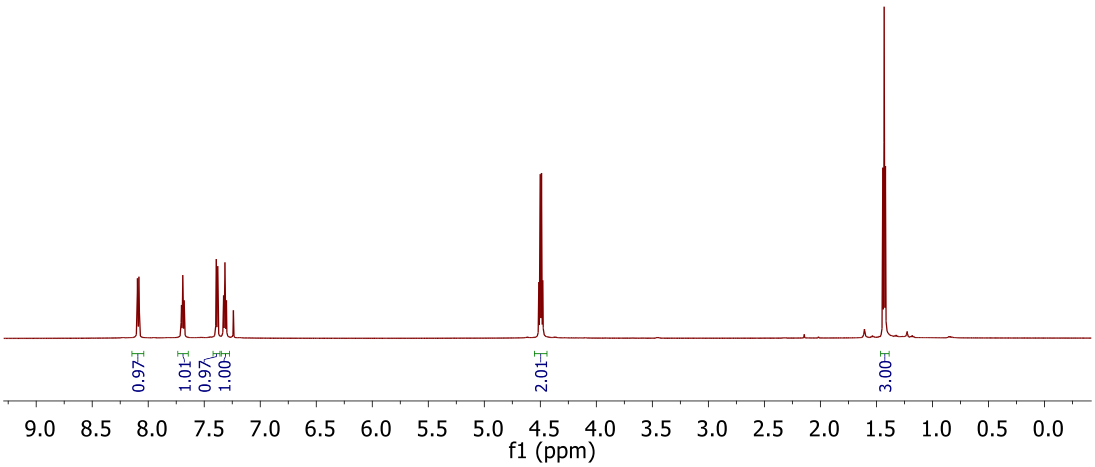


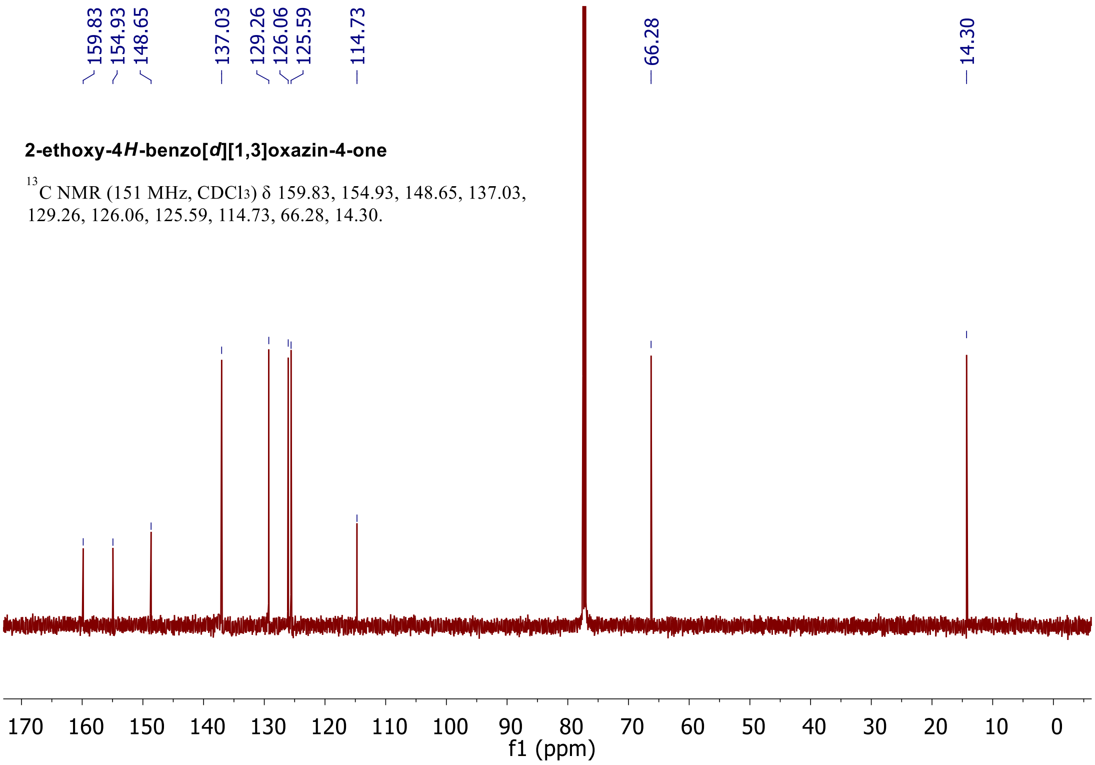


**2-ethoxy-4*H*-benzo[*d*][1,3]oxazin-4-one**

$^{13}$C NMR (151 MHz, CDCl3) δ 159.83, 154.93, 148.65, 137.03, 129.26, 126.06, 125.59, 114.73, 66.28, 14.30.

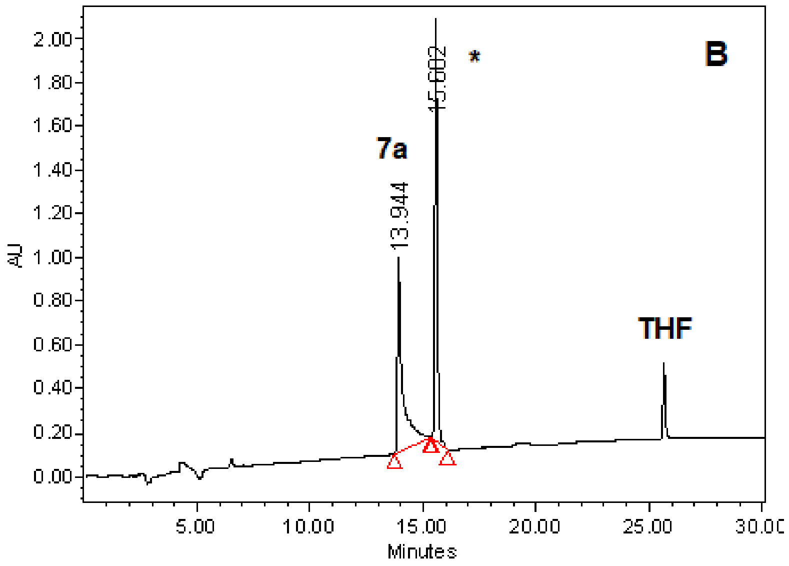


**Figure S24: (A)** $^{1}$H-NMR and $^{13}$C-NMR of 2-ethoxy-4*H*-benzo[*d*][1,3]oxazin-4-one: $^{1}$H NMR (600 MHz, $CDCl_3$) δ 8.09 (dd, *J* = 7.9, 1.2 Hz, 1H), 7.74 – 7.64 (m, 1H), 7.39 (d, *J* = 8.1 Hz, 1H), 7.31 (dd, *J* = 11.2, 3.9 Hz, 1H), 4.50 (q, *J* = 7.1 Hz, 2H), 1.43 (t, *J* = 7.1 Hz, 3H); $^{13}$C NMR (151 MHz, $CDCl_3$) δ 159.83, 154.93, 148.65, 137.03, 129.26, 126.06, 125.59, 114.73, 66.28, 14.30; **(B)** Hplc analysis of 2-ethoxy-3,1-benzoxazin-4-one (*). Hplc column/conditions: Puroshpere RP-8 (5µm); 250mm–4mm; 1.0 ml/min; 20% to 100% AcCN in 30 min; Abs: 214 nm.

## H) HPLC analysis of the reaction of

## 2-ethoxy-4*H*-benzo[*d*][1,3]oxazin-4-one (*) with LiCl/LiBr

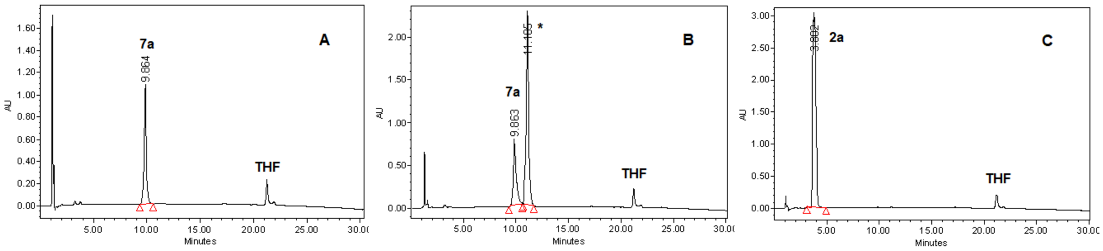


**Figure S25:** Hplc analysis of: **(A) 7a**; **(B) 2-ethoxy-3,1-benzoxazin-4-one (*)**; **(C) 2a**. Hplc column/conditions: Lichrosphere RP-8e (5µm); 125mm–4mm; 1.0 ml/min; 20% to 100% AcCN in 30 min; Abs: 214 nm.

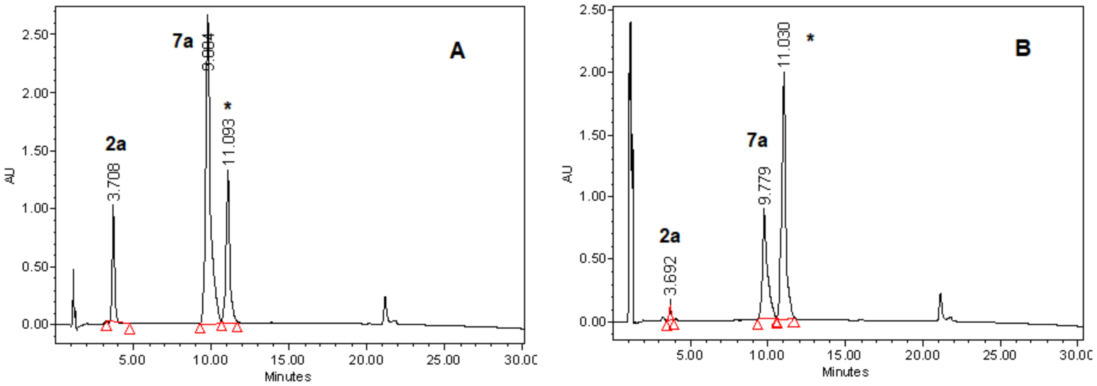


**Figure S26:** Hplc analysis of: **(A)** the reaction of **2-ethoxy-3,1-benzoxazin-4-one with LiCl in THF** (0.2 M) at room temperature (22–24 °C) at 24 h; **(B)** the reaction of **2-ethoxy-3,1-benzoxazin-4-one with LiBr in THF** (0.2 M) at room temperature (22–24 °C) at 24 h. Hplc column/conditions: Lichrosphere RP-8e (5µm); 125mm–4mm; 1.0 ml/min; 20% to 100% AcCN in 30 min; Abs: 214 nm.

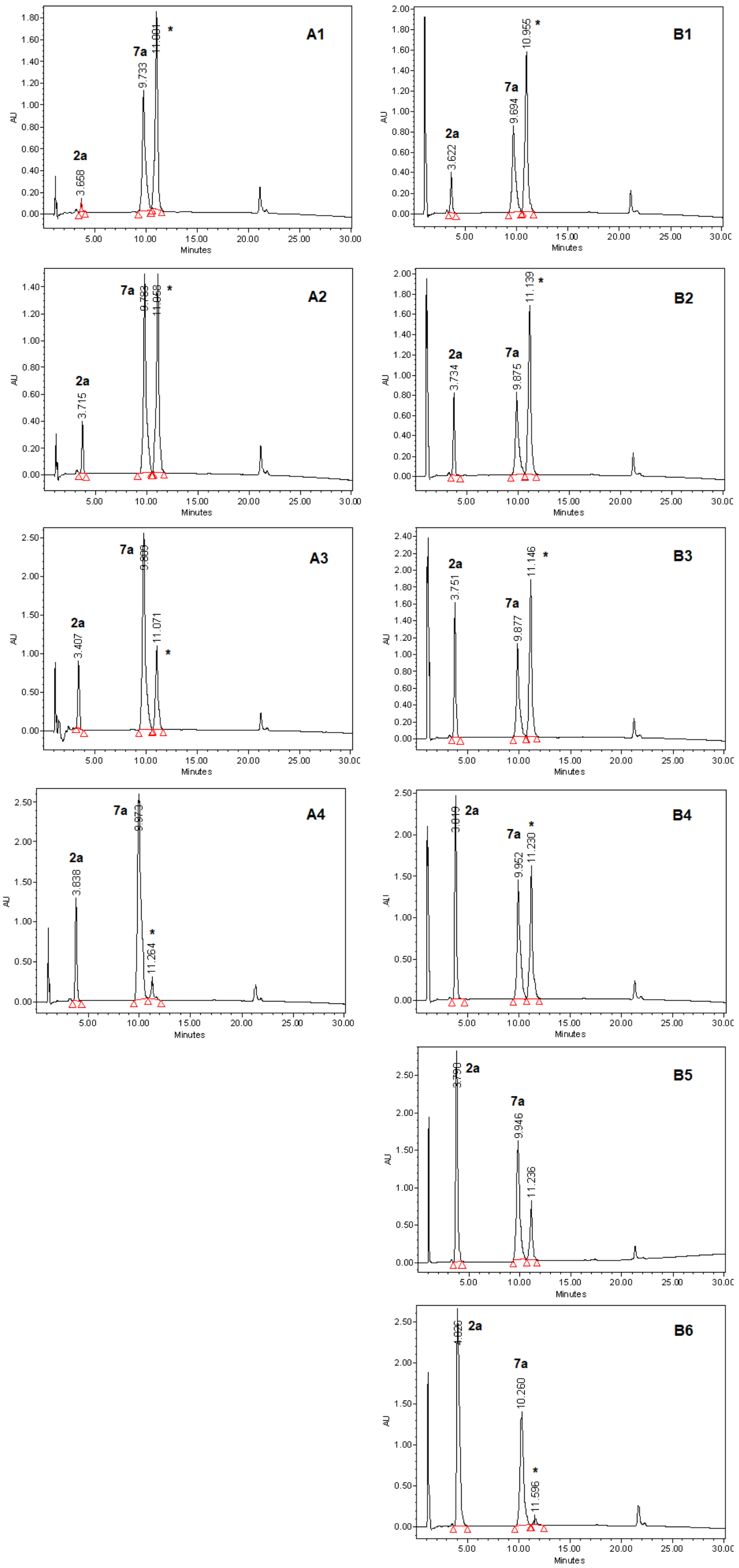


**Figure S27: (A1–4)** Hplc analysis of the reaction of **2-ethoxy-3,1-benzoxazin-4-one with LiCl in THF/1% AcOH** (0.2 M) at room temperature (22–24 ºC) at 2 h (A1), 12 h (A2), 24 h (A3), 48 h (A4); **(B1–4)** Hplc analysis of the reaction of **2-ethoxy-3,1-benzoxazin-4-one with LiBr in THF/1% AcOH** (0.2 M) at room temperature (22–24 ºC) at 2 h (B1), 12 h (B2), 24 h (B3), 48 h (B4), 3 d (B5), 5 d (B6). Hplc column/conditions: Lichrosphere RP-8e (5µm); 125mm–4mm; 1.0 ml/min; 20% to 100% AcCN in 30 min; Abs: 214 nm.

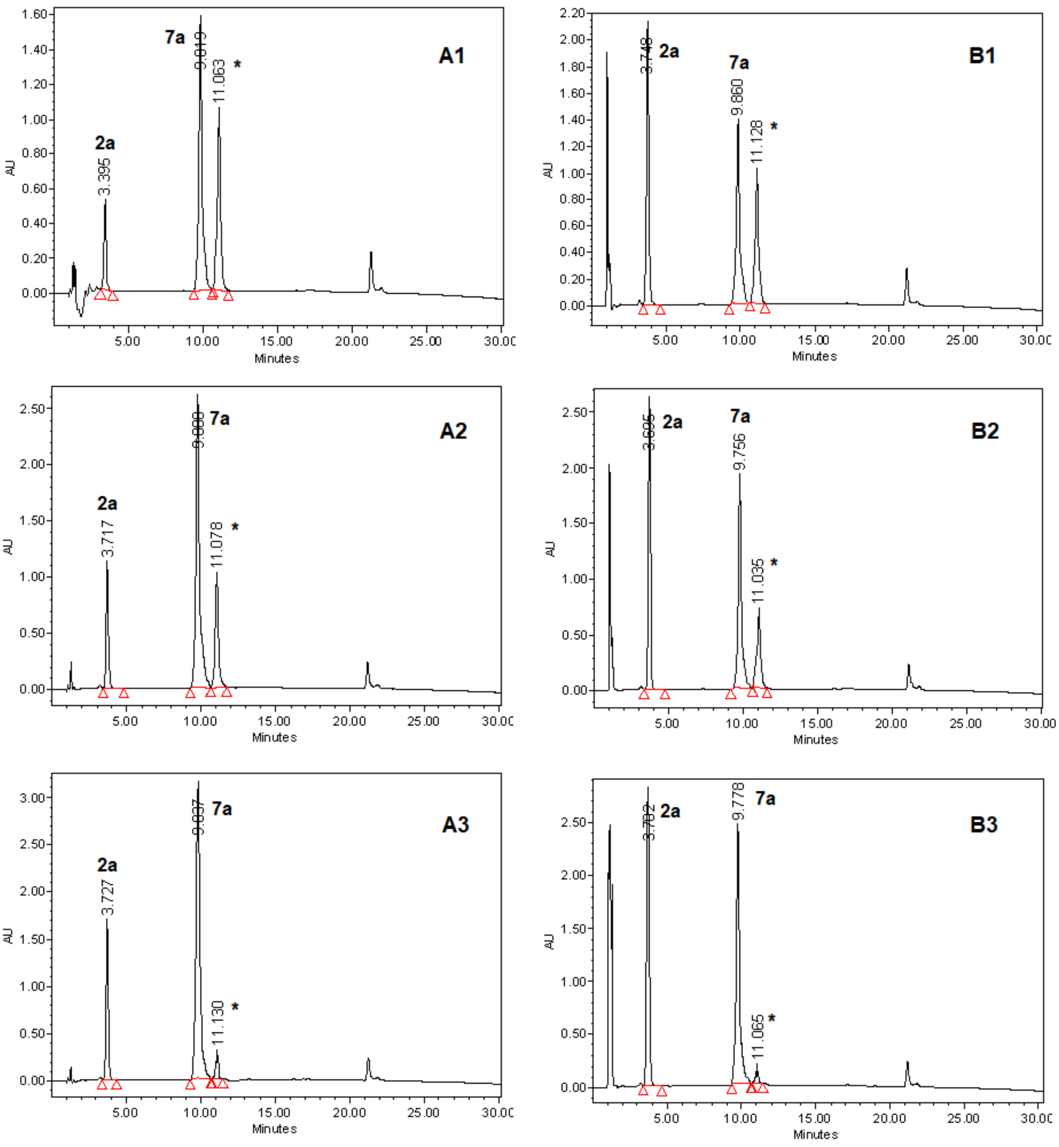


**Figure S28: (A1–3)** Hplc analysis of the reaction of **2-ethoxy-3,1-benzoxazin-4-one with LiCl in THF/10% AcOH** (0.2 M) at room temperature (22–24 °C) at 12 h (A1), 24 h (A2), 48 h (A3); **(B1–3)** Hplc analysis of the reaction of **2-ethoxy-3,1-benzoxazin-4-one with LiBr in THF/10% AcOH** (0.2 M) at room temperature (22–24 °C) at 12 h (B1), 24 h (B2), 48 h (B3). Hplc column/conditions: Lichrosphere RP-8e (5µm); 125mm–4mm; 1.0 ml/min; 20% to 100% AcCN in 30 min; Abs: 214 nm.

## References

[37] H. F. Koch, W. C. Pomerantz, E. L. Ruggles, M. Van Laren, A.-M. Van Roon, *Collect. Czech. Chem. Commun.*, 2002, **67**, 1505–1516.
